# Fault diagnosis of a CNC hobbing cutter through machine learning using three axis vibration data

**DOI:** 10.1016/j.heliyon.2025.e41637

**Published:** 2025-01-07

**Authors:** Nagesh Tambake, Bhagyesh Deshmukh, Sujit Pardeshi, Sachin Salunkhe, Robert Cep, Emad Abouel Nasr

**Affiliations:** a Department of Mechanical Engineering, Walchand Institute of Technology, Solapur, Maharashtra, India; b Department of Mechanical Engineering, COEP Technological University, Pune, Maharashtra, India; c Department of Biosciences, Saveetha School of Engineering, Saveetha Institute of Medical and Technical Sciences, Chennai, India; d Gazi University Faculty of Engineering, Department of Mechanical Engineering, Maltepe, ANKARA, Turkey; e Department of Machining, Assembly and Engineering Metrology, Faculty of Mechanical Engineering, VSB-Technical University of Ostrava, 17. Listopadu 2172/15, 708 00, Ostrava, Czech Republic; f Department of Industrial Engineering, College of Engineering, King Saud University, PO Box 800, Riyadh, 11421, Saudi Arabia

**Keywords:** CNC hobbing cutter, Machine learning, Fault diagnosis, Vibration data, Feature engineering, Ensemble Model, Explainable AI

## Abstract

This research presents a novel approach to fault diagnosis for CNC hobbing cutters using machine learning techniques, leveraging three-axis vibration data to ensure machining precision and tool reliability. Traditional methods of tool monitoring are insufficient for real-time and complex machining environments, prompting the integration of automated machine learning models. A robust dataset was collected from a CNC hobbing machine, capturing vibration signals under healthy and faulty tool conditions. Statistical features, including Root Mean Square (RMS), Crest Factor, and Kurtosis, were extracted from the vibration data for model training. Various machine learning algorithms, including Decision Trees, Efficient Linear models, Neural Networks, and Ensemble methods, were evaluated for their classification accuracy. Among these, the Ensemble model achieved perfect classification accuracy (100 %) with minimal computational cost, making it optimal for real-time applications. Explainable AI techniques, such as LIME and Shapley values, were employed to interpret model predictions, enhancing the system's transparency and reliability. The proposed framework demonstrated superior performance compared to existing methodologies in the literature, addressing key gaps such as overfitting, data quality, and model explainability. Real-world deployment challenges, including diverse operating conditions and generalizability across machines, were also discussed, with recommendations for incorporating multi-sensor data and transfer learning approaches in future research. This study establishes a foundation for predictive maintenance in CNC machining, significantly reducing downtime and improving operational efficiency through precise fault diagnosis in hobbing cutters.

## 1 Introduction

In the machining industry, ensuring the health of cutting tools is paramount to achieving high-quality products, efficient production, and prolonged machine tool lifespan. Traditional fault diagnosis methods, such as visual inspection and basic sensor readings, are often subjective and limited, making them unsuitable for complex, high-speed machining environments. Consequently, there is a growing interest in robust, automated fault-diagnosis methods, with machine learning (ML) emerging as a promising solution. By leveraging sensor data, ML algorithms can accurately identify and classify cutting tool faults, enabling real-time and precise monitoring [1]. Among the sensor data sources, tri-axial vibration data collected from accelerometers mounted on the tool or machine body provides valuable insights into machining dynamics. These vibrations reflect the influence of cutting forces, tool geometry, material properties, and tool health, exhibiting significant variations under fault conditions such as excessive wear, chipped edges, or improper installation. By analyzing these patterns using ML algorithms, automated fault diagnosis becomes feasible [2]. The ML-based fault diagnosis process typically involves three stages: data collection, feature engineering, and model training. During the data collection phase, tri-axial vibration data is captured continuously or at specific intervals, depending on the monitoring strategy. For accurate fault detection, the data acquisition system must encompass a broad frequency range to capture relevant machining dynamics [3]. Feature engineering, the next step, extracts essential characteristics from the raw vibration signals. Time-domain metrics (e.g., mean, standard deviation), frequency-domain features (e.g., Root Mean Square (RMS), centroid, peak frequencies), and time-frequency domain features derived from advanced techniques like wavelet transforms provide a comprehensive representation of signal behavior [4]. Proper feature selection enhances fault classification accuracy by focusing on informative data [5]. A variety of ML algorithms have demonstrated success in fault diagnosis, including Support Vector Machines (SVMs), K-nearest neighbors (KNNs), Random Forests, and Artificial Neural Networks (ANNs) [6]. Recently, advanced techniques such as Modified Kernel Extreme Learning Machines (MKELMs), Kernel Entropy Component Analysis (KECA), and One-Against-One Least Squares Support Vector Machines (OAO LSSVM) have shown significant promise in enhancing diagnostic accuracy [7].

Moreover, methodologies like curvilinear Manhattan distance evaluation and voltage difference analysis have been used to refine fault classification further by improving feature relevance and interpretability [8,9]. Wavelet-based feature extraction is another critical area of innovation. Selecting an optimal wavelet basis function enhances the sensitivity and specificity of fault detection. For example, combining wavelet transforms with MKELM can yield precise and computationally efficient fault diagnosis systems, especially in non-stationary machining environments [10]. Deep learning approaches, including Convolutional Neural Networks (CNNs) and Recurrent Neural Networks (RNNs), have also transformed the field by automating feature extraction and learning intricate patterns from raw vibration data [11]. Transfer learning further accelerates the deployment of ML models by adapting pre-trained networks for new datasets, reducing computational effort and data requirements [12]. While integrating ML in cutting tool health monitoring offers real-time insights, consistency, and scalability, it faces challenges. Developing labeled datasets that cover diverse machining conditions is time-intensive and expensive. The non-stationary nature of vibration signals due to progressive tool wear adds complexity to fault detection. Real-world implementation must address constraints such as real-time computation, optimal sensor placement, and user-friendly visualization of diagnostic results [13]. Innovative strategies such as ensemble modeling, simulation-based training, and dimensionality reduction techniques are addressing these challenges. For instance, combining KECA with OAO LSSVM has been shown to enhance fault detection accuracy, while voltage difference analysis aids in distinguishing subtle fault conditions [14,15]. Advances in simulation tools reduce reliance on real-world data by generating diverse fault scenarios, enabling models to generalize better to unseen conditions [16]. These developments underscore the transformative impact of ML on fault diagnosis, paving the way for more efficient and accurate cutting tool monitoring in machining applications. However, there is a need to address the following research gaps, particularly in the application of ML to specific tools like hobbing cutters, to realize its full potential.

• *Lack of Research on Hobbing Machines:* While tool condition monitoring for conventional lathes and milling machines has been extensively studied, there is a noticeable gap in fault prediction specifically for CNC hobbing machines using three-axis vibration data. This research aims to fill this void.
• *Data Quality and Diversity:* Existing studies often suffer from limited, low-quality, or non-representative data, which impacts model generalization. High-quality, standardized datasets encompassing diverse machining scenarios and fault types for hobbing cutters are scarce.
• *Overfitting Issues in ML Models:* Many current studies face challenges like overfitting, where models perform well on training data but fail to generalize to unseen data. This issue is particularly prevalent when the models are not optimized for real-world variations.
• *Explainability and Interpretability:* Previous research lacks focus on the interpretability of ML models used for fault diagnosis, making it difficult for end-users to trust and understand the system's decisions.
• *Feature Engineering and Selection:* Most prior studies rely on either time-domain or frequency-domain features in isolation. A comprehensive approach that combines statistical and time-frequency features is not well-explored.

The research specifically targets and contributes to these identified shortcomings by.

• Providing a robust dataset of three-axis vibration data for hobbing cutters.
• Utilizing advanced ML techniques like Bayesian optimization to prevent overfitting.
• Applying XAI for enhanced transparency and reliability.

## 2 Methodology

**Fig. 1:**
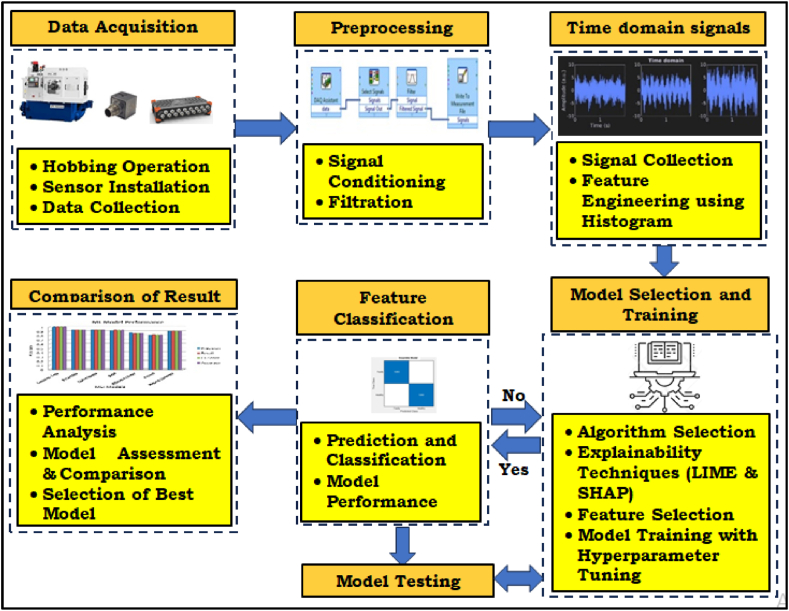
Methodology Flowchart.

Fig. 1 is showing, the methodology for fault diagnosis of a CNC hobbing cutter, the process begins with data acquisition, where vibration data were collected from three axes during the hobbing operation using installed sensors. This raw data was then preprocessed, involving signal conditioning and filtration to remove noise and enhance signal quality. The preprocessed data was transformed into time-domain signals, and feature engineering techniques, such as histogram analysis, were applied to extract relevant features. Next, the extracted features are used for feature classification, where the data is categorized into different fault classes.

Model selection and training follow, where various machine learning algorithms are evaluated and the best model is selected and trained on the feature-rich dataset. The trained model is then tested on unseen data to assess its performance and ensure generalization. To enhance model interpretability, explainability techniques like LIME and SHAP were employed to understand the factors contributing to the model's predictions. Feature selection was performed to identify the most informative features, leading to improved model performance. Hyperparameter tuning was conducted to optimize the model's parameters for better results. Finally, the trained and optimized model was used for the prediction and classification of new data, enabling real-time fault diagnosis of the CNC hobbing cutter.

## 3 Experimental setup

**Fig. 2:**
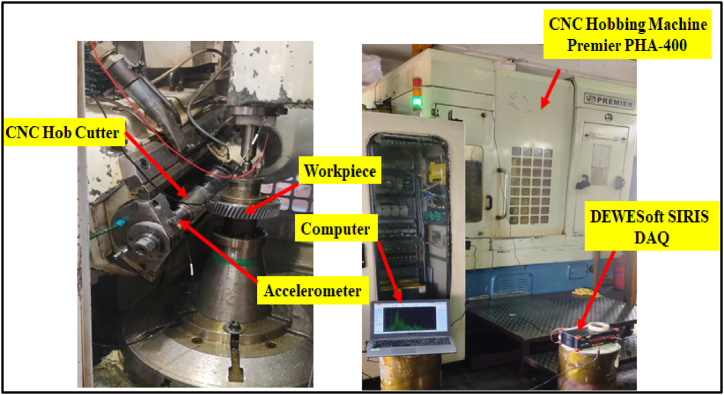
Set up of experiment.

The experiments were conducted under controlled conditions with uniform machining parameters, and the coolant flow and type remained consistent throughout the experiments. In this scenario, variations in vibration signals due to coolant were minimal and not a significant factor in the fault diagnosis model. The experiment for collecting vibration data was conducted at Laxmi Hydraulics Pvt. Ltd. in Solapur, utilizing a CNC Hobbing Machine (Premier PHA-400), as depicted in Fig. 2. A sensitive vibration sensor, specifically an Integrated Electronics Piezo Electric (IEPE) tri-axial accelerometer sensor with a sensitivity of 10 mV/g, was positioned near the frame of the machine's spindle. This is a common practice because vibrations are typically most pronounced near moving parts. In CNC machining, any parameter changes initially impact the cutting tool, which then transmits vibrations to the spindle due to its horizontal rotation. Adhering to established practices [17], the accelerometer was positioned vertically to align with the spindle's axis of rotation.

**Fig. 3:**
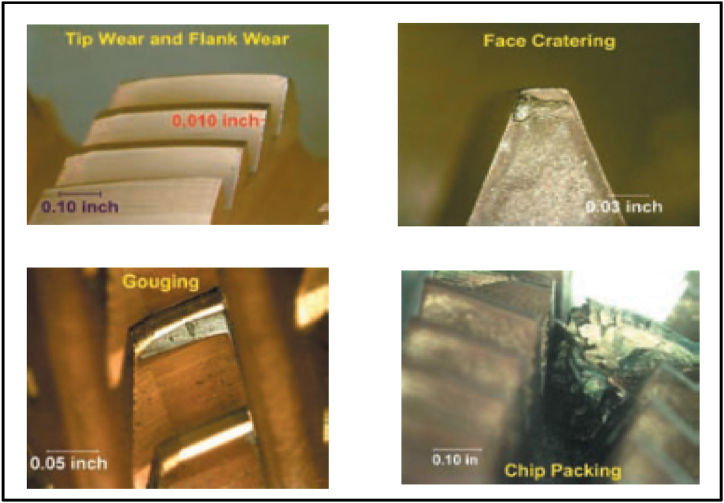
Hob Cutter Faults [18].

Fig. 3 illustrates common hobbing cutter faults, with tip and flank wear being the most frequent issues. This wear can gradually eat through the tool's coating, exposing the hob material underneath. Additionally, craters may develop on the hob's surface. If these craters become severe enough to reach the cutting edge, they can cause the entire hobbing tool to fail. The details of hob cutter faults are elaborated [18].

• *Edge Chipping:* This can happen on the top, sides, or both, depending on the tool material's suitability for the job. It can be caused by the material being too brittle, too hard or vibrations during cutting.
• *Built-Up Edge (BUE):* This occurs when workpiece material sticks to the cutting tool. This is common with soft materials like copper alloys and can be caused by improper coolant, insufficient coolant flow, or inadequate tool clearance.
• *Chip Packing and Shelling:* When a lot of material is removed and there's not enough space for chips to evacuate, they can pack between the hob teeth. This can lead to teeth breaking off, called shelling.
• *Other Failures:* Grinding cracks from tool sharpening or microchipping can also cause hob failure.

To monitor tool conditions, “DewesoftX” software was used to actively capture and record a real-time graph depicting the relationship between time (in seconds) and acceleration (measured in g-forces). Vibration signals were collected during gear hobbing operations performed on a 20MnCr5 case-hardened gear blank, using an 80 mm diameter and 120 mm length high-speed steel (HSS) hobbing cutter with a 2.75 mm module. The machining parameters remained uniform throughout all machining processes, featuring a 6.8 mm depth of cut, 40 rpm spindle speed, and 0.8 mm/min feed rate. Two distinct machining conditions were executed, maintaining the depth of cut, feed and the speed constant and recording vibration signals. Details on these conditions are shown in Table 1.

**Table 1:**
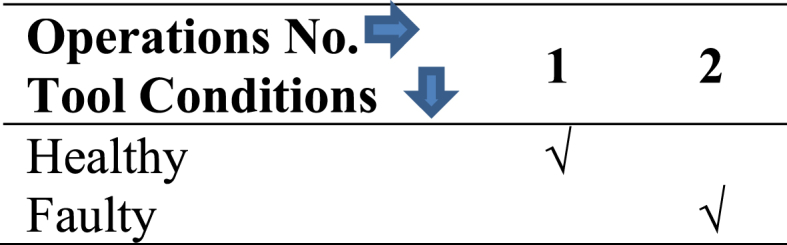
Two conditions of hobbing Cutter.

## 4 Data collection of signals

**Fig. 4:**
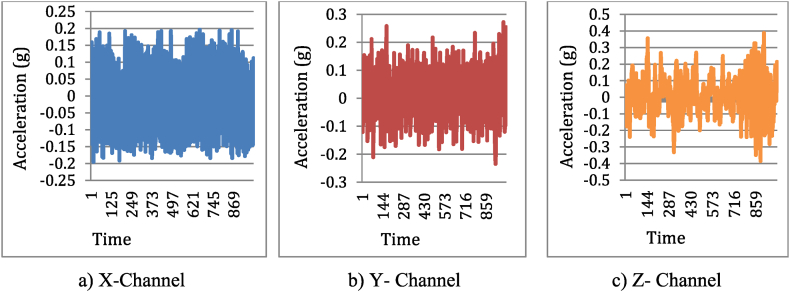
Time domain signals of healthy hobbing cutter.

**Fig. 5:**
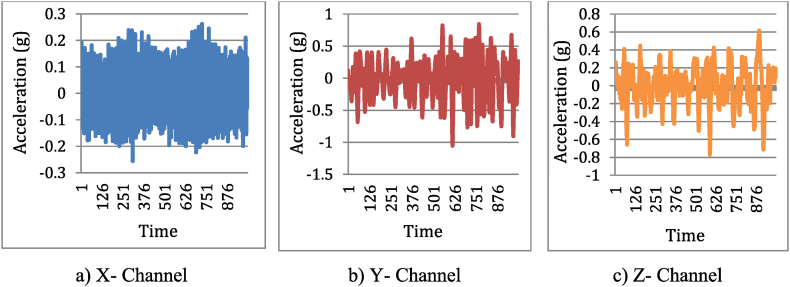
Time domain signals of faulty hobbing cutter.

To get accurate results, experiments require a careful selection of processing settings. These settings, like sampling rate in signal recording, how many times a machine learning algorithm repeats its training, and classifier hyperparameters, significantly affect data collection, feature extraction, and model training. Picking the wrong settings can throw off the entire experiment's findings. The rationale for choosing a sampling rate of 40.1 kHz in this experiment is primarily based on the Nyquist theorem, which dictates that the sampling rate should be at least twice the highest frequency present in the signal to avoid aliasing [19]. During the experiment, the highest frequency was recorded as 4010 Hz. Ensuring that the vibration signals are accurately captured. Vibration signals were captured for both machining operations using “DewesoftX software”, which generated time-series graphs (Fig. 3). Each operation lasted 20 s, and we collected 802000 data points per operation. Initially, a machining step prepared the workpiece by removing imperfections and oxide layers, achieving a stable state. As indicated by Fig. 4, Fig. 5, around 1000 readings of the vibration signal's acceleration amplitude (measured by Dewesoft SIRIUS) exhibit a trend of increasing values as the hobbing cutter condition deteriorates. The time domain signals for healthy and faulty hobbing cutters appear to differ across all three channels (X, Y, and Z).

However, the faulty hobbing cutter signals have higher amplitude compared to the healthy hobbing cutter signals. The signal oscillates between a range of approximately 0.15 g to −0.15 g for all three channels (X, Y, and Z). The signal is a sinusoidal wave, indicating a periodic and relatively smooth vibration. The signal oscillates between a higher range of approximately 0.25 g to −0.25 g for all three channels (X, Y, and Z).

This discrepancy was attributed to the directional nature of the vibration data captured by the tri-axial accelerometer. Each channel (X, Y, Z) measures vibrations along a different axis (horizontal, vertical, and axial directions, respectively). The variations in amplitude between healthy and faulty conditions are influenced by how the faults propagate vibrations in different directions due to the dynamic forces involved.

### 4.1 Physical reasons for changes in the vibration signals

Changes in vibration signals across the X, Y, and Z axes in a CNC hobbing cutter arise from machining forces and fault impacts, with each axis representing distinct directional vibrations: X (horizontal), Y (vertical), and Z (axial along the spindle).

• *X-axis (horizontal):* Vibrations reflect lateral cutting forces, subtly influenced by faults like flank wear or edge chipping, which cause irregular contact and force variations depending on machining conditions.
• *Y-axis (vertical):* Vertical vibrations decrease with faults such as tip wear or microchipping, indicating reduced cutting efficiency and redistribution of forces towards the Z-axis.
• *Z-axis (axial):* Vibrations increase significantly with faults like edge chipping or tip wear, reflecting instability and force irregularities along the spindle's axis.

Analyzing these directional vibrations reveals how faults uniquely affect each axis, offering a detailed understanding of the tool's health and dynamic force changes.

## 5 Machine learning technique and its performance evaluation

### 5.1 Feature engineering

The data-driven method used in MATLAB for feature extraction was a statistical approach. This step uses histograms to graphically represent how often certain values appear among the 8 statistical features extracted from the CNC hobbing cutter's vibration data. These features are [20].

• *Peak value:* Largest magnitude of the signal.

$$Peak\;Value\;\left(a_{p}\right)=\max\;{ǀa}_{i}ǀ$$

• *Crest Factor:* The ratio between a signal’s highest point (peak) and its root mean square (RMS) is known as the crest factor. Changes in a signal’s peakiness can often indicate developing faults before they become evident in the overall energy of the signal (RMS). Therefore, the crest factor can serve as an early warning system for detecting faults.

$$Crest\;Factor=\frac{a_{p}}{\frac{1}{n}\sum_{j=1}^{n}{a_{i}}^{2}}$$

• *Kurtosis:* Kurtosis is a measure of how extreme values (outliers) are present in a signal’s distribution. Think of it like the length of the signal’s tails - a higher kurtosis indicates more outliers. Faults in the system can cause more outliers, which would be reflected by an increase in the kurtosis value. In a normal distribution, kurtosis typically equals 3.

$$Kurtosis=\frac{\frac{1}{n}\sum_{j=1}^{n}{\left(a_{i}-a\right)}^{4}}{{\left(\frac{1}{n}\sum_{j=1}^{n}{\left(a_{j}-a\right)}^{2}\right)}^{2}}-3$$

• *Root Mean Square (RMS):* The average of the values obtained by squaring each number in the set.

$$Root\;Mean\;Square=\sqrt{\frac{\sum_{i=1}^{N}a_{i}^{2}}{N}}$$

• *Standard Deviation (Std.):* This statistic measures how scattered the data is around its central point (mean).

$$Standard\;Deviation=\sqrt{\frac{\sum_{i=1}^{n}{\left(a_{i}-a\right)}^{2}}{n-1}}$$

• *Skewness:* An uneven spread of signals can occur. When problems arise, the distribution becomes lopsided, and this is reflected by a higher skewness value.

$$Skewness=\frac{\frac{1}{n}\sum_{j=1}^{n}{\left(a_{j}-a\right)}^{2}}{{\left(\frac{1}{n}\sum_{j=1}^{n}{\left(a_{j}-a\right)}^{2}\right)}^{\frac{1}{2}}}$$

$${Where,a}_{i},i=1,\ldots \ldots ,n\;sample\;sensor\;data\text{.}$$

• *Signal to Noise and Distortion Ratio (SINAD):* Proportion of total signal strength relative to the combined noise and distortion power.
• *Signal-to-Noise Ratio (SNR):* Strength of the signal compared to the background noise.
• *Total Harmonic Distortion (THD):* Proportion of distortion power to ideal power.

Statistical features were extracted using histograms to analyse 12,830 samples (6415 healthy and 6415 faulty) of CNC hobbing cutter data. Four key features per channel were derived, with histograms graphically representing these features to reveal patterns distinguishing healthy from faulty cutters. This balanced dataset and diverse features enabled the identification of unique characteristics crucial for reliable fault diagnosis. The repetition of features allowed a detailed examination, enhancing accuracy in fault detection and improving maintenance practices. This statistical approach effectively separated healthy and faulty conditions, aiding in the development of robust diagnostic systems.

**Fig. 6:**
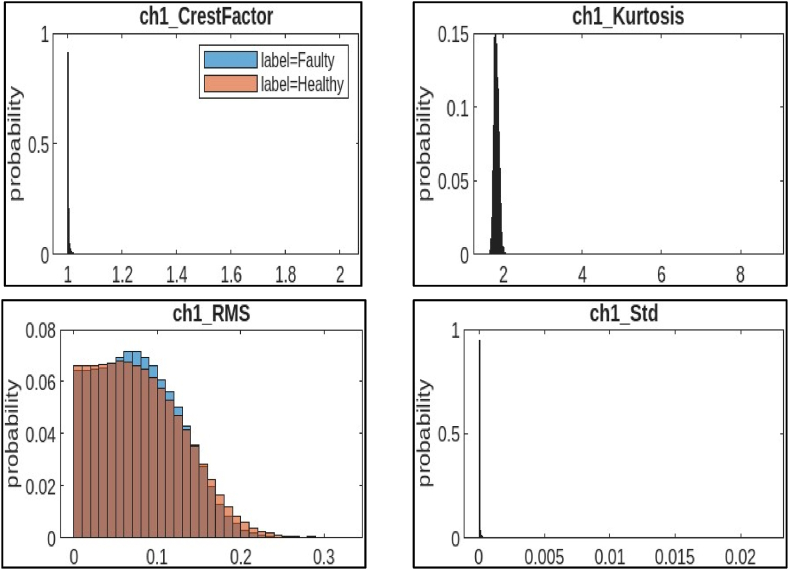
Histogram features of channel 1.
b) Channel 2 (ch2): Fig. 7 shows four histograms that visualize the distribution of statistical features extracted from channel 2 of a CNC hobbing cutter.
  ● *Mean:* It is the average of all the values in a dataset. The histogram shows that the mean values in channel 2 are concentrated around 0.2, with a lower probability of values outside this range. The distribution seems to be centered around a specific mean value, with data points tapering off on either side.
  ● *RMS:* It is a statistical measure of the amplitude of a signal. The histogram shows that the RMS values in channel 2 are also concentrated around 0.2, with a lower probability of values outside this range. The distribution looks similar to ch1 RMS, with a rightward skew and more data points concentrated on lower values.
  ● *Skewness:* It is a measure of how symmetrical a distribution is. When the data leans to the right in a histogram, it's called a positive skew. Conversely, if it leans to the left, it's considered a negative skew. The histogram shows that the skewness values in channel 2 are spread out between −2 and 3, with a peak around 1. This suggests that the distribution of skewness values is slightly skewed to the right.
  ● *Std.:* It is a measure of the spread of the data from the mean. A higher standard deviation indicates that the data is more spread out. The histogram shows that the standard deviation values in channel 2 are spread out between 0 and 0.3, with a peak around 0.1. The distribution appears somewhat symmetrical, possibly normally distributed around a central standard deviation value.

a) Channel 1 (ch1)**:** Fig. 6 shows four histograms that visualize the distribution of statistical features extracted from channel 1 of a CNC hobbing cutter. These features were calculated from vibration signals collected from the machine. In machine learning-based fault diagnosis systems, these features are used to train a model to identify faults in the cutter.
  ● *Crest Factor:* It is a measure of the “peakiness” of a signal. A higher crest factor indicates a sharper peak relative to the average of the signal. The histogram shows that most of the crest factor values in channel 1 fall between 1.02 and 1.06, with a lower probability of values outside this range. This suggests that most crest factor values fall within a specific range, but there are some instances with significantly higher values.
  ● *Kurtosis:* It is a measure of how the tails of a distribution differ from those of a normal distribution. A distribution with a higher kurtosis than a normal distribution is called “leptokurtic” and has fatter tails. The histogram shows that the kurtosis values in channel 1 tend to be clustered around 2, with a slight skew towards lower values. This suggests that the distribution of kurtosis values is close to normal.
  ● *RMS:* It is a statistical measure of the amplitude of a signal. The histogram shows that the RMS values in channel 1 are concentrated around 0.1, with a lower probability of values outside this range. The distribution leans towards the left, with more data points concentrated on the lower end and a fatter tail towards higher RMS values.
  ● *Std.:* It is a measure of how spread out the data is from the mean. A higher standard deviation indicates that the data is more spread out. The histogram shows that the standard deviation values in channel 1 are spread out between 0 and 0.02, with a peak around 0.01. The distribution appears to be bell-shaped, centered on a certain standard deviation value.

**Fig. 7:**
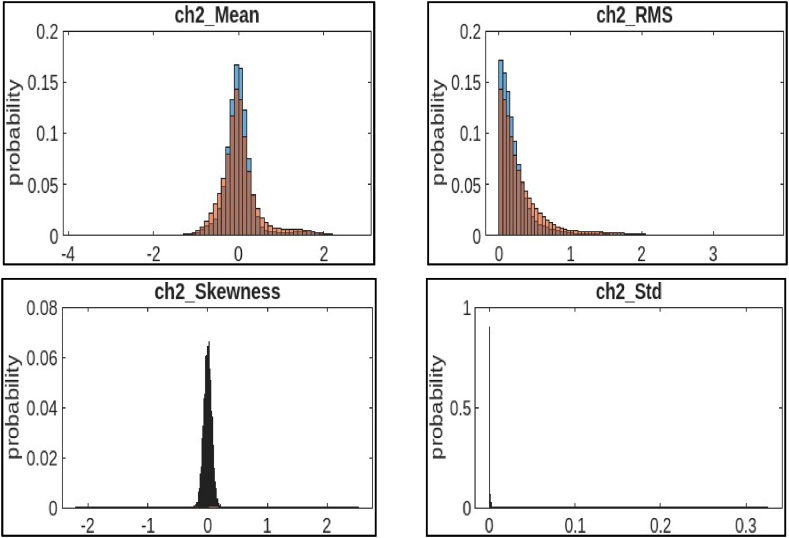
Histogram features of channel 2.

**Fig. 8:**
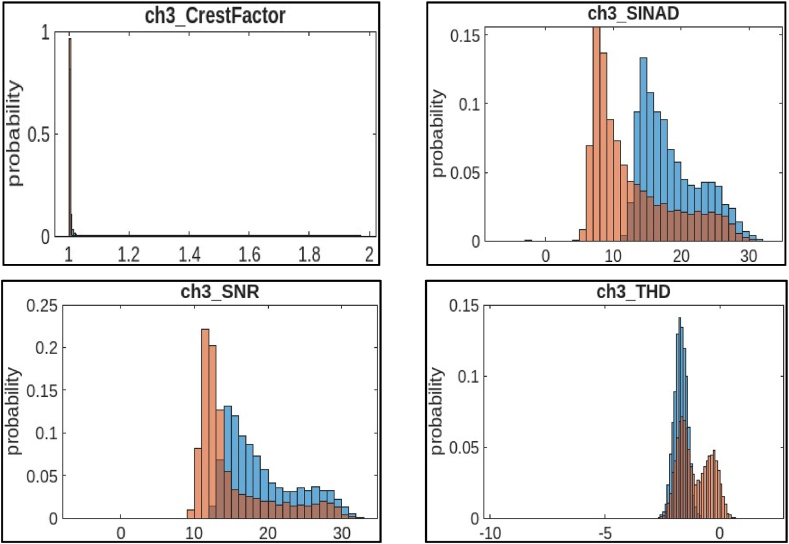
Histogram features of channel 3.
c) **Channel 3 (ch3):**Fig. 8 shows four histograms that visualize the distribution of statistical features extracted from channel 2 of a CNC hobbing cutter.
  ● *Crest Factor:* It is a measure of the “peakiness” of a signal. A higher crest factor indicates a sharper peak relative to the average of the signal. The histogram shows that most of the crest factor values in channel 3 fall between 1.2 and 1.4, with a lower probability of values outside this range. The distribution appears similar to ch1 crest factor, with a skew towards higher values.
  ● *SINAD:* It is a rating that indicates the clarity of a signal. It compares the strength of the main signal to the scrambled noise and distortions that can be present. A higher SINAD ratio indicates a stronger signal relative to noise and distortion. The histogram shows that most of the SINAD values in channel 3 fall between 0.1 and 0.15, with a lower probability of values outside this range.
  ● *SNR:* It is similar to SINAD but doesn't take into account distortion. The histogram shows that most of the SNR values in channel 3 fall between 0 and 0.1, with a lower probability of values outside this range. The distribution leans rightward, with more data concentrated on lower SNR values and a tail toward higher values.
  ● *THD:* It is a metric that gauges the amount of unwanted impurities that have infiltrated a signal. A higher THD value indicates a more distorted signal. The histogram shows that most of the THD values in channel 3 fall between 0 and 0.05, with a lower probability of values outside this range. It appears wider than a normal distribution.

The observed distribution patterns indicated potential variations in the signal quality and noise levels, which are crucial factors in fault diagnosis. It implies different operating conditions or potential issues with the CNC hobbing cutter that need to be further investigated for optimal performance and maintenance. These features were used as inputs to a machine learning model along with labels indicating the data was collected during the healthy or faulty operation of the cutter. The model would then be trained to identify features that correlate with faults. Once trained, the model was used to classify new data points as healthy or faulty.

### 5.2 Model selection and training

#### 5.2.1 Rationale for choosing specific machine learning techniques for model training

Choosing the best machine learning algorithms for classifying features in CNC hobbing cutter health monitoring requires a thoughtful approach. The following are the reasons why the selected three algorithms are suitable for this application.

• *Decision trees:* These handle both numerical and categorical data, which might be present in CNC hobbing cutter features. Additionally, they are interpretable, allowing us to understand how the model arrives at decisions related to tool health [21].
• *Efficient Linear:* If the features have a straight-forward impact on tool health, techniques like linear regression or logistic regression can work well. Kernel algorithms like kernel SVM are powerful for capturing non-linear relationships in the data [22,23].
• *Ensemble methods:* By combining multiple models, ensemble methods like Random Forest or AdaBoost improve the model's overall strength and ability to generalize to unseen data, leading to more accurate predictions [24].
• *Deep Neural Network:* Neural networks, particularly deep learning models, are effective for feature classification in condition monitoring due to their ability to learn complex patterns in data. For CNC hobbing cutters, these models can process intricate sensor data and identify subtle patterns missed by traditional algorithms. This is crucial for predictive maintenance, allowing early detection of issues to prevent downtime and extend equipment life. Deep learning can accurately recognize patterns linked to tool wear, misalignment, and other faults, enabling timely interventions [25].

The selection of algorithms prioritized those capable of effectively dealing with the inherent characteristics of CNC machine sensor data, such as high dimensionality, non-linear relationships, and real-time processing demands. Performance evaluation will utilize various metrics including accuracy, precision, recall, and F1 score to enable a comparative assessment.

#### 5.2.2 Optimization of hyperparameters for training the models

a) **Process of Hyperparameter tuning:** In machine learning, iterations and hyperparameters are critical for training algorithms, affecting learning, model complexity, and generalization. Poor parameter choices can lead to overfitting, where the model memorizes data, or underfitting, where it misses patterns, resulting in poor performance. Bayesian optimization, a data-driven technique, is used for hyperparameter tuning, improving model performance and reducing computational costs [26]. This approach fine-tunes decision tree models for classifying tool conditions using vibration data to assess tool health states effectively.
  • *Data Loading:* First, the system reads vibration data from an Excel spreadsheet. This data consists of various statistical features, where each column represents a specific feature. Then, the data is carefully separated into categories based on the type of tool wear, such as healthy, chipped teeth, or different wear patterns on the tip and flank.
  • *Feature and Label Integration:* To prepare the data for the model, two key components are created: a features matrix (X) and a labels matrix (Y). The X matrix brings together all the statistical features extracted from the different tool wear categories. The Y matrix, on the other hand, holds corresponding labels that indicate the specific wear condition for each data point.
  • *Decision Tree Construction:* The system utilizes machine learning algorithms to construct the models. These algorithms, like fitclinear, fitcensemble, fitctree and net are specifically designed to learn from the data and identify patterns that can be used for classification.
  • *Model:* This model is constructed with hyperparameters automatically optimized using the ‘OptimizeHyperparameters’,’auto’ options.

This process outlines the steps involved in using vibration data for tool condition assessment. It covers how to import the data, transform it for machine learning analysis, and build models by fine-tuning their internal settings to achieve the best performance. The results obtained in section 5.3.2, based on the procedure outlined above.

**Fig. 9:**
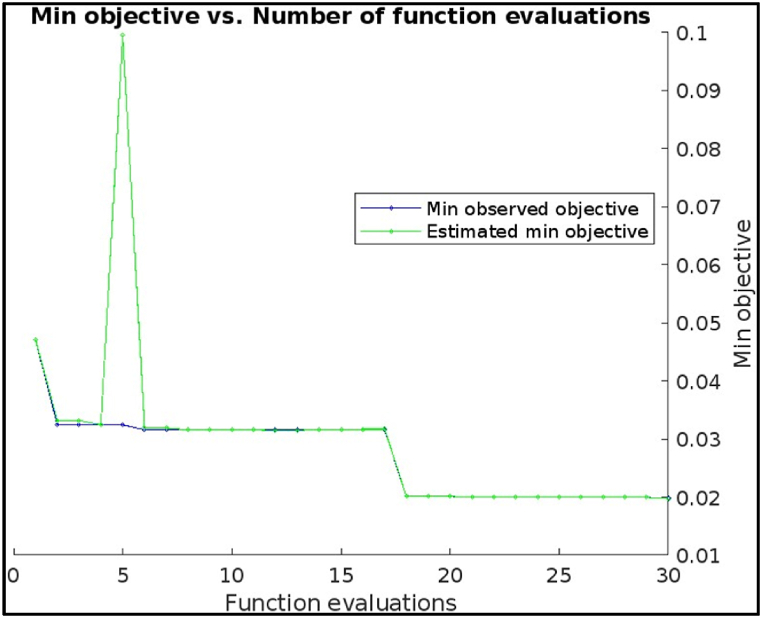
Minimum objective vs. Number of functions evaluations plot of Efficient Linear Model.

b) Hyperparameter Tuning for Classification Tasks: Hyperparameter tuning is the process of adjusting the settings of a machine learning model to achieve the best possible performance. In this case, The Selected ML algorithms were trained using 80 % data and the models were trying to minimize a specific objective function. The horizontal axis (x-axis) tracks the number of times the model assessed its performance (function evaluations) during the hyperparameter tuning process. The vertical axis (y-axis) shows the results of these evaluations. Lower values on the y-axis indicate better model performance.
  i) Efficient Linear: Fig. 9 shows the results of hyperparameter tuning for an efficient linear model used in Fault Diagnosis of a CNC hobbing cutter. The hyperparameters selected by Bayesian optimization technique for efficient linear algorithms are:
    ● *Regularization Strength (Lambda):* This value controls the amount of regularization applied by the ridge regularization technique. To avoid overfitting, ridge regression introduces a penalty that discourages models from becoming too intricate. The chosen value (3.3225e-04) seems to be effective in this case, as the algorithm was able to learn from the data without overfitting.
    ● *Learner (SVM):* This approach leverages a Support Vector Machine (SVM) for classification. SVMs were particularly adept at tackling intricate categorization problems and perform well even when dealing with data containing many features with high-dimensional data.
    ● *Regularization (Ridge):* Ridge regression works by incorporating an additional penalty into the model's learning process (cost function). This penalty discourages overly complex models that might lead to overfitting. This penalty term discourages the learning algorithm from assigning excessively large weights to the features. This step strengthens the model's generalizability, allowing it to deliver reliable results on unseen data.

Fig. 9 indicates, the Minimum objective vs. Number of functions evaluations plot for hyperparameter tuning using the Bayesian optimization method of an Efficient Linear Model. The plot shows that the minimum objective function value has not significantly decreased as the number of function evaluations increased. This suggests that the Bayesian optimization method may not be finding much improvement in the Efficient Linear Model's performance with the current hyperparameter settings. The minimum observed objective value (around 0.08) stagnates after roughly 10 function evaluations. This indicates that the Bayesian optimization wasn't efficiently exploring better hyperparameter configurations. The following result indicates that the optimization process was stopped early at 30 function evaluations, possibly due to reaching the maximum evaluations limit. However, given the limited improvement observed, stopping earlier might have been reasonable.

**Figure:**
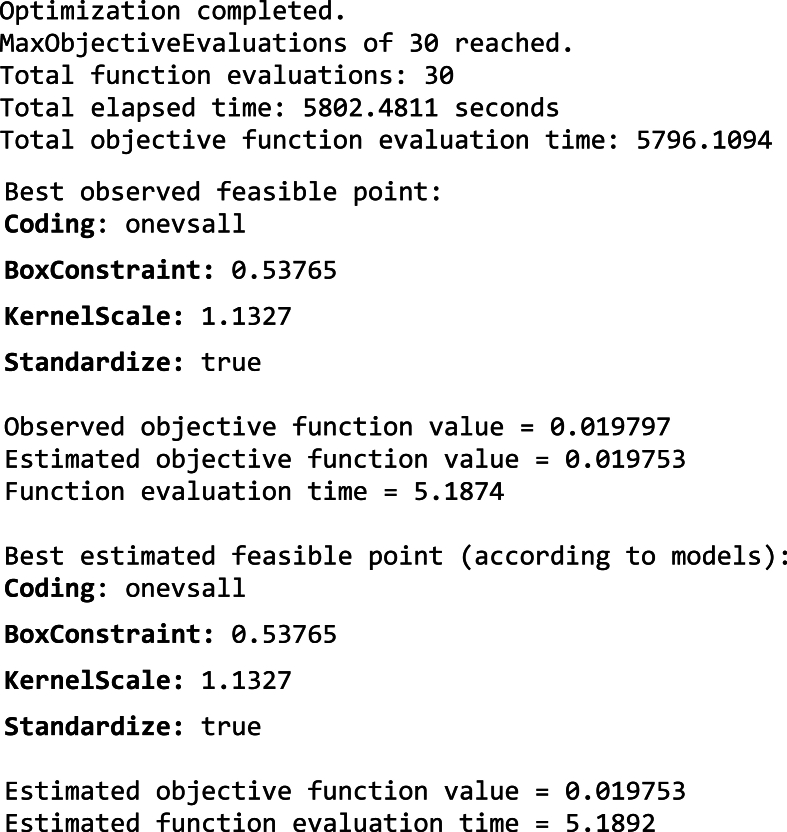

**Fig. 10:**
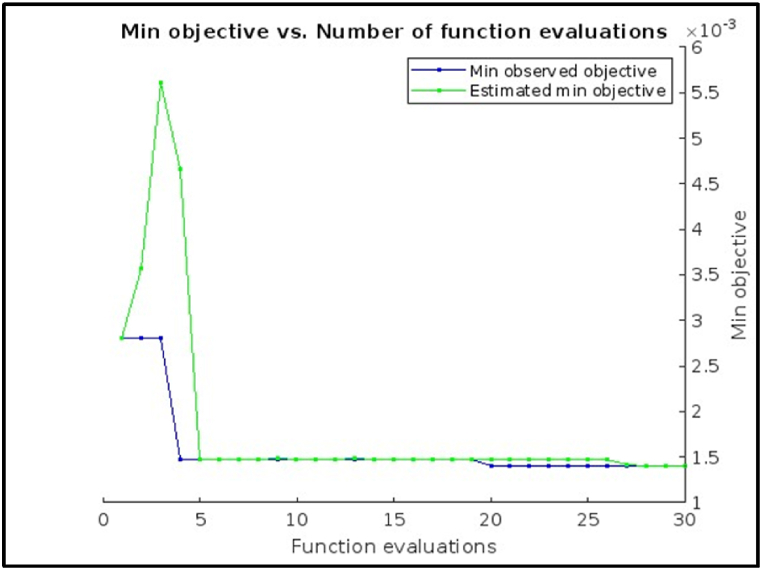
Minimum objective vs. Number of functions evaluations plot of Decision Tree Model.

The above plot result suggests that the Bayesian optimization method was not well-suited for tuning the Efficient Linear Model in this case.

ii) Decision Tree: Fig. 10 shows the results of hyperparameter tuning for a Decision Tree model used in Fault Diagnosis of a CNC hobbing cutter. The hyperparameters selected by Bayesian optimization technique for decision tree algorithms are:
  ● *Maximum number of splits (131):* This hyperparameter controls the maximum depth of the decision tree. A higher value allows the tree to grow more complex, potentially improving its ability to fit the training data. However, a very deep tree leads to overfitting. In this case, 131 seems to be a sufficient value to capture the patterns in the data without overfitting.
  ● *Split criterion: Maximum deviance reduction:* This criterion determines how the decision tree chooses the best split at each node. The maximum deviance reduction criterion selects the split that maximizes the reduction in the deviance (mean squared error) at that node. This strategy helps to improve the purity of the leaves in the decision tree, which leads to better classification performance.

**Fig. 11:**
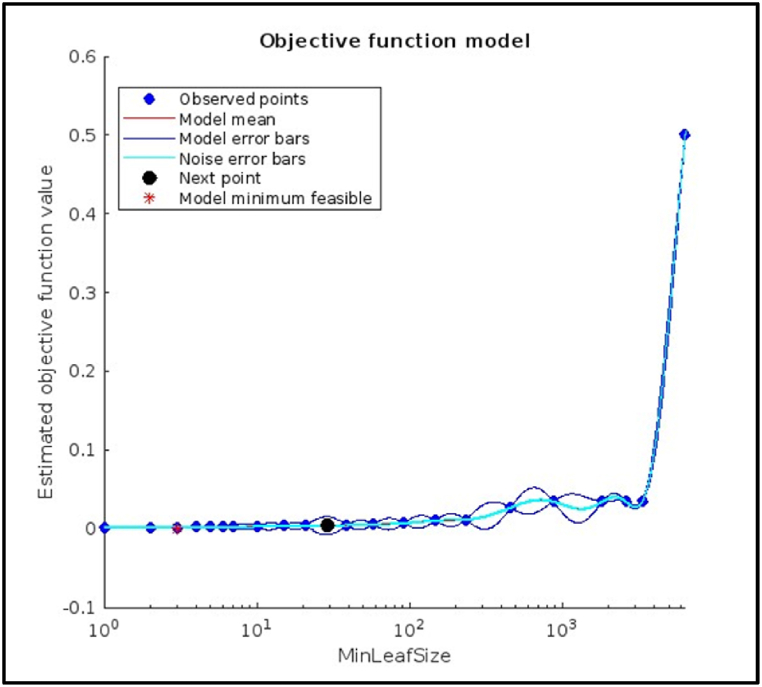
Objective function model plot of Decision Tree Model.

Fig. 11 which shows plots for hyperparameter tuning using the Bayesian optimization method of a Decision Tree Model for Fault Diagnosis of a CNC hobbing cutter using Machine Learning, here are the key observations: The plot shows a trend of decreasing minimum objective value as the number of function evaluations increases. This suggests that the Bayesian optimization method was effectively identifying better hyperparameter combinations for the decision tree model. Even though there is a downward trend, the rate of improvement appears to be slowing down after 25 function evaluations. There might still be room for improvement, but it likely requires more evaluations than performed in this case (which is 30). Fig. 11 shows that the objective function value (represented by color intensity) is sensitive to the value of the hyperparameter MinLeafSize. Lower MinLeafSize values (darker blue) tend to correspond to lower objective function values, indicating better model performance. It shows following result.

**Figure:**
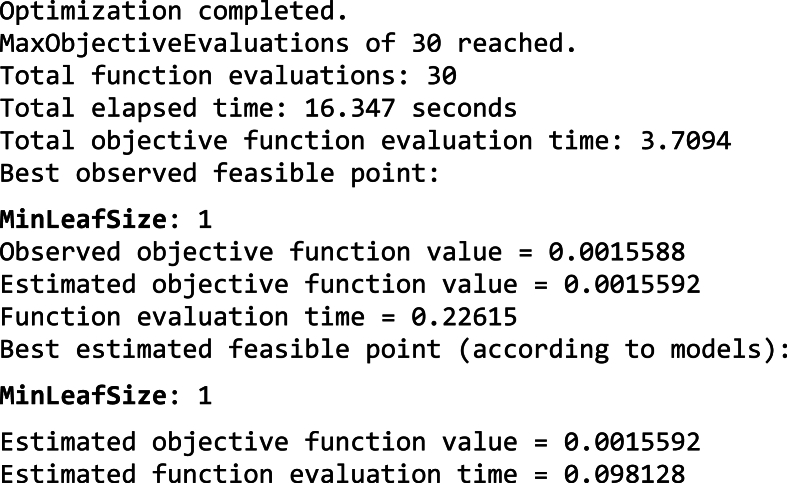

The above plot result suggests that the Bayesian optimization method was making some progress in tuning the decision tree model. However, the improvement was slow and the stopping point might not have been optimal.

iii) **Ensemble:**Fig. 10 shows the results of hyperparameter tuning for an efficient linear model used in Fault Diagnosis of a CNC hobbing cutter. The specific ensemble method used was bagging, with the following hyperparameters selected by Bayesian optimization technique are:
● *Number of learners (10):* This ensemble method uses ten individual classifiers (likely decision trees) to make predictions.
● *Maximum number of splits (660):* This parameter controls the maximum number of times the training data splits into subsets for training the individual learners. A higher value allows for more diverse learners, which will potentially improve the overall performance of the ensemble.
● *Number of predictors to sample (6):* This hyperparameter specifies the number of features to randomly sample from the total set of features for training each individual learner in the ensemble. This process, called feature bagging, helps to reduce variance and prevent overfitting.

**Fig. 12:**
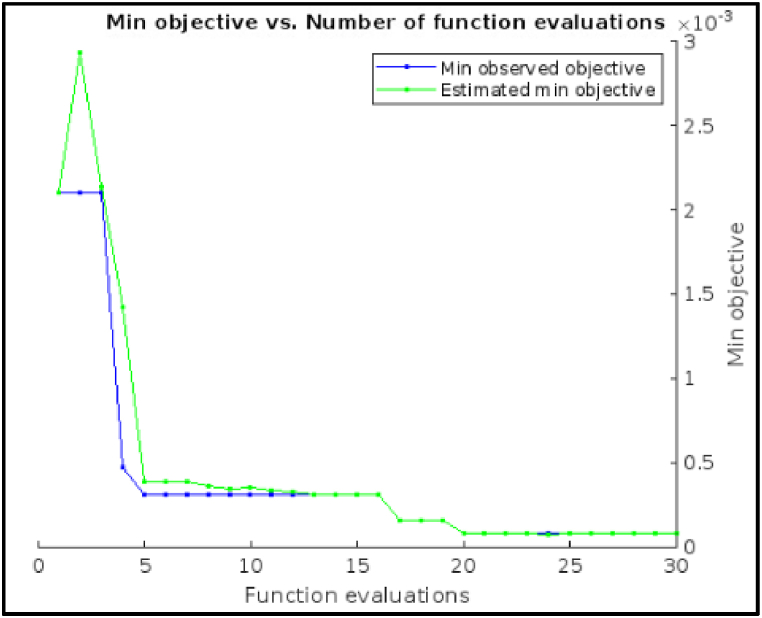
Minimum objective vs. Number of functions evaluations plot of Ensemble Model.

Fig. 12 shows, the Minimum objective vs. Number of functions evaluations plot for hyperparameter tuning using the Bayesian optimization method of an Ensemble Model. The minimum observed objective function value (around 0.078) decreases steadily as the number of function evaluations increases. This suggests that the Bayesian optimization method is effectively identifying promising hyperparameter configurations to evaluate and refine the ensemble model.

The plot shows that the rate of improvement in the minimum objective function value starts to slow down after about 20 function evaluations. This could indicate that a good stopping point for the hyperparameter tuning process might be around that point. It's important to consider the trade-off between further refinement and computational cost when making this decision. The following result indicates that the optimization process was stopped early, at 30 function evaluations, likely due to reaching the maximum evaluations limit.

**Figure:**
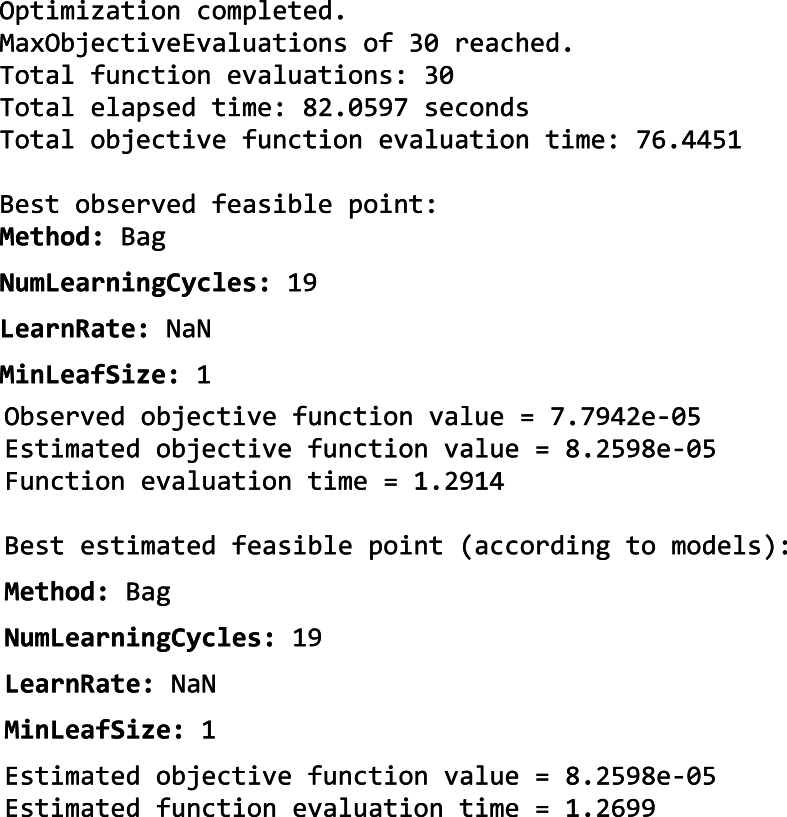

The above plot result suggests that the Bayesian optimization method was working well to improve the Ensemble Model's performance. Even though the optimization was terminated early, the minimum objective function value achieved was promising.

iv) **Deep Neural Network:** The hyperparameter tuning results for the Deep Neural Network model used in fault diagnosis of a CNC hobbing cutter can be interpreted as follows:
  • *Number of Fully Connected Layers (1):* This indicates the model has a single fully connected (dense) layer. A single layer simplifies the architecture, potentially reducing overfitting and computational complexity while ensuring sufficient learning capacity for the dataset's features.
  • *Activation (ReLU):* The Rectified Linear Unit (ReLU) activation function introduces non-linearity into the model, enabling it to learn complex patterns in the data. ReLU is computationally efficient and avoids issues like vanishing gradients, making it well-suited for fault diagnosis tasks.
  • *Standardize Data (Yes):* Standardization ensures the input data has a mean of 0 and a standard deviation of 1. This normalization improves model convergence during training by maintaining numerical stability and ensuring uniform feature scaling.
  • *Regularization Strength (Lambda) (2.6247e-05):* This value specifies the regularization term in the loss function, which penalizes large weights to prevent overfitting. A small value, such as 2.6247e-05, indicates a light penalty, balancing model flexibility and overfitting prevention.
  • *First Layer Size (183):* The first layer has 183 neurons, which aligns with the dimensionality of the input features or is determined to capture sufficient information for the subsequent layer. This size is tuned to achieve optimal performance for fault classification tasks.

This tuned configuration strikes a balance between model complexity and generalization, ensuring accurate fault diagnosis while minimizing overfitting and computational overhead.

**Fig. 13:**
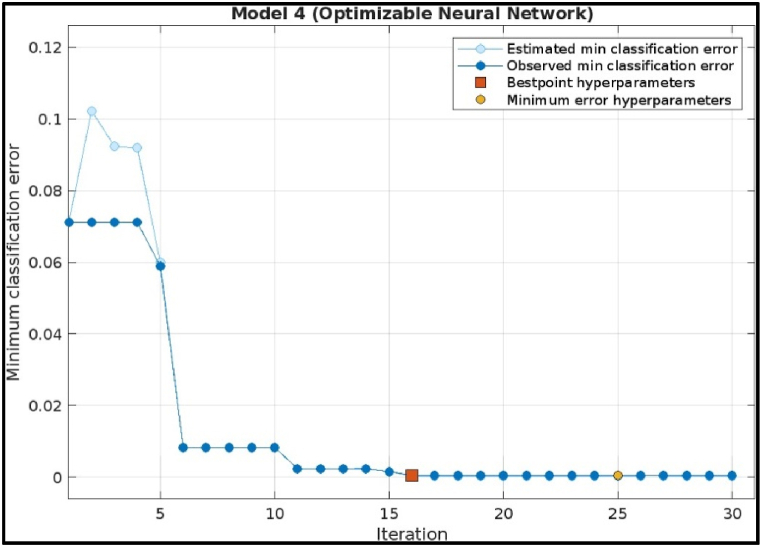
Minimum classification error plot of Deep Neural Network Model.

Fig. 13 shows the hyperparameter tuning process for a Deep Neural Network used in fault diagnosis of a CNC hobbing cutter. The x-axis represents the number of iterations, while the y-axis indicates the minimum classification error. Initially, both the estimated and observed classification errors are relatively high but decrease significantly after around the 5th iteration, indicating that better hyperparameters are being identified. The best point hyperparameters (red square) and the minimum error hyperparameters (yellow circle) mark the iterations where the model achieves optimal and minimal classification errors, respectively. The plot suggests that the tuning successfully minimized classification error, enhancing the model's accuracy.

**Fig. 14:**
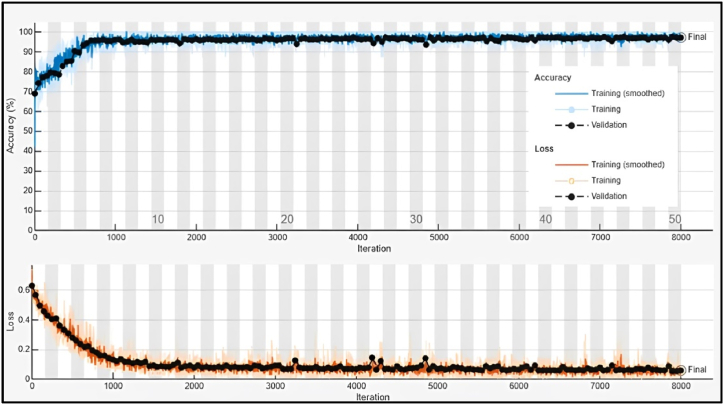
Training and validation performance of Deep Neural Network Model.

Fig. 14 illustrates the training and validation performance of the Deep Neural Network model used for fault diagnosis in a CNC hobbing cutter. In the accuracy plot (top), both training and validation accuracies start low but increase steadily, stabilizing near 99.94 % after approximately 4000 iterations, indicating excellent learning and minimal overfitting. In the loss plot (bottom), the training and validation losses decrease sharply during the initial iterations and stabilize near 0.06, reflecting effective convergence. The smooth trends in both accuracy and loss demonstrate that the model has been optimized well, achieving high reliability for fault classification.

#### 5.2.3 Identifying the most effective machine learning model via a well-tuned hyperparameter search

Based on the results in Section 5.2.2, the Ensemble Model proved to be the most effective for fault diagnosis of CNC hobbing cutters among the four models evaluated (Efficient Linear, Decision Tree, Ensemble, and Deep Neural Network). Despite incremental improvements, the Efficient Linear Model showed limitations for complex scenarios. The Decision Tree Model provided interpretability and effective fault classification but lacked robustness in datasets with overlapping features. The Ensemble Model, optimized through bagging and hyperparameter tuning, demonstrated superior generalization, adaptability, and flawless performance in diverse fault scenarios, making it ideal for real-time fault diagnosis. The Deep Neural Network, while accurate, was constrained by high resource demands, limiting its practicality. The Ensemble Model's exceptional accuracy, efficiency, and reliability underscore its suitability for predictive maintenance in CNC machining environments.

### 5.3 Feature importance using explainable AI (XAI) techniques

Both LIME (Local Interpretable Model-Agnostic Explanations) and Shapley values are techniques used in Explainable AI (XAI) to explain the inner workings of a black box model. That is, they attempt to explain how each feature influences the model's prediction. Fig. 13, Fig. 14 shows, the LIME and Shapley values for each feature used in a Ensemble model to diagnose the fault status of a CNC hobbing cutter. In both LIME and Shapley value techniques, a higher positive value indicates a greater contribution to the prediction of a healthy cutter, while a higher negative value indicates a greater contribution to the prediction of a faulty cutter [27].

• *LIME Values:* LIME works by introducing small perturbations to a single data instance and seeing how the model's prediction changes. Algorithm 1 outlines the steps for an algorithm that uses LIME to explain a classifier's predictions for a particular instance in an Ensemble model.

Algorithm 1 **Classifier's predictions for a particular instance using LIME**

**Figure:**
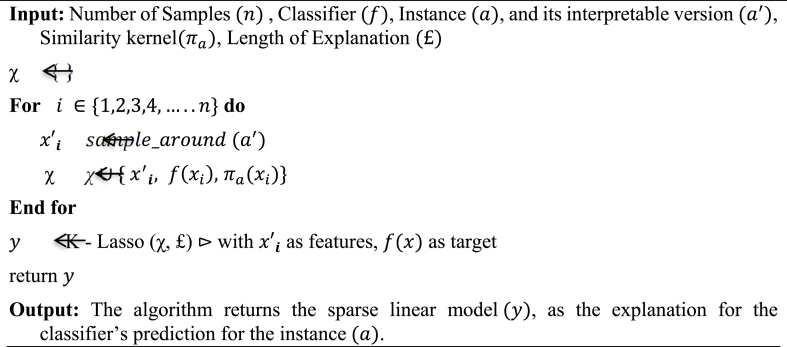

**Fig. 15:**
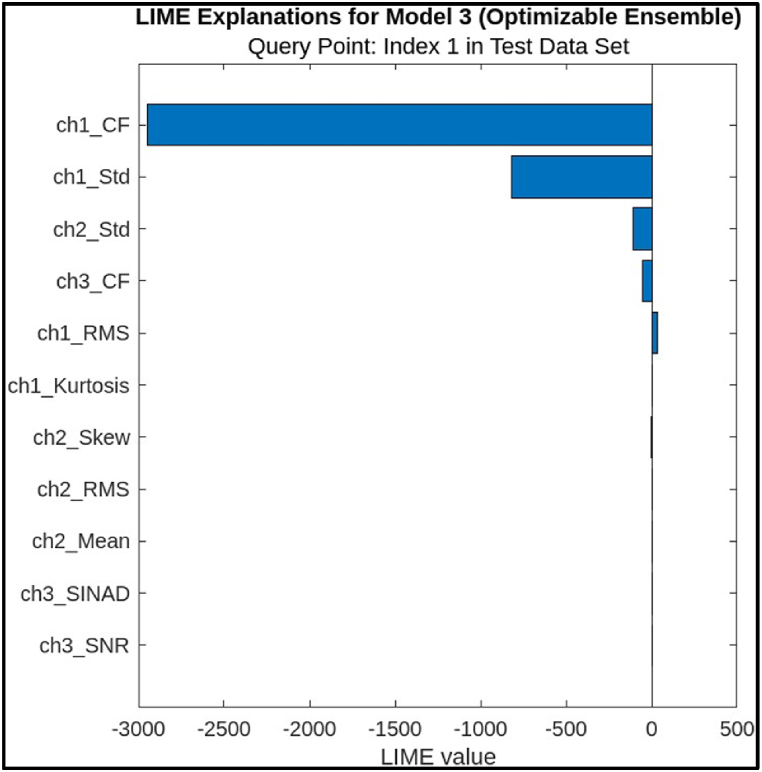
Lime Explanations for ensemble Model.

The bar chart, Fig. 15, visually depicts the LIME values for each predictor, with the x-axis representing the LIME value and the y-axis enumerating the predictors. A negative LIME value indicates that a decrease in the predictor's value would likely lead to a decrease in the model's prediction for the query point, suggesting the predictor's importance in the diagnosis. Conversely, a positive LIME value implies that an increase in the predictor could potentially increase the model's prediction. Table 2, provides a numerical representation of the same information, listing each predictor, its corresponding value for the query point (Index 1 in the test dataset), and its associated LIME value.

**Table 2:** Predictor & LIME Value of features.

Predictor	Predictor Value	LIME Value
ch1_CF	1.0101	−2947.7
ch1_Std	0.0015477	−816.0053
ch2_Std	0.01505	−106.0678
ch3_CF	1.0078	−52.5629
ch1_RMS	0.2675	35.818
ch1_Kurtosis	1.7454	1.1127
ch2_Skew	−0.014738	−0.916
ch2_RMS	3.0043	0.2358
ch2_Mean	−3.0043	0.0842
ch3_SINAD	23.766	0.0249
ch3_SNR	27.7775	0.0113

From Tables 2 and it is evident that the most influential predictor in this instance is ‘ch1_CF’ with a substantial negative LIME value of −2947.7. This implies that a reduction in the ‘ch1_CF’ value would significantly decrease the model's prediction of a fault. Other notable predictors with considerable negative LIME values include ‘ch1_Std’, ‘ch2_Std’, and ‘ch3_CF’, suggesting their substantial impact on the model's decision-making process. In contrast, predictors like ‘ch1_RMS’, ‘ch1_Kurtosis’, and ‘ch2_RMS’ exhibit positive LIME values, indicating that an increase in their values might potentially elevate the model's prediction of a fault. However, the magnitude of these positive values is considerably smaller compared to the negative values, implying a lesser influence on the overall prediction.

• Shapley Values: Shapley values are based on the concept of fair allocation of credit among a group of cooperating players. In this case, the “players” are the features, and the “game” is the machine learning model's prediction. Algorithm 2 outlines an algorithm estimating a feature's contribution to a model's output, based on a simplified Shapley value approach in an Ensemble model.

**Table:** 

Algorithm 2: Estimating a feature's contribution to a model's output, based on a simplified Shapley value approach
**Input:** ML Model (M), training set (A), feature index (i), Instance of interest (a), Number of iterations (N) **Output:** Shapley value for the feature value (i) **For**n=1,2,3,4,……N**do** Draw random instance y from the dataset Z Order instance $a:{\;a}_{0}=\left(a_{1},\ldots \ldots a_{i}\ldots \ldots \ldots a_{p}\right)$ Order instance $y:{\;y}_{0}=\left(y_{1},\ldots \ldots y_{i}\ldots \ldots \ldots y_{p}\right)$ Construct two new instances With $i:a_{+i}=\left(a_{1},\ldots \ldots a_{i-1}\ldots \ldots \ldots a_{p},y_{i+1},\ldots \ldots y_{p}\right)$ Without $i:a_{-i}=\left(a_{1},\ldots \ldots a_{i-1}\ldots \ldots \ldots a_{p},y_{i+1},\ldots \ldots y_{p}\right)$ Compute marginal contribution: $\emptyset_{i}^{n}=M\left(a_{+i}\right)-M\left(a_{-i}\right)$ **End for** Compute Shapley value as the average: $\emptyset_{i}\left(a\right)=\frac{1}{N}\sum_{n=1}^{N}\emptyset_{i}^{n}$

**Fig. 16:**
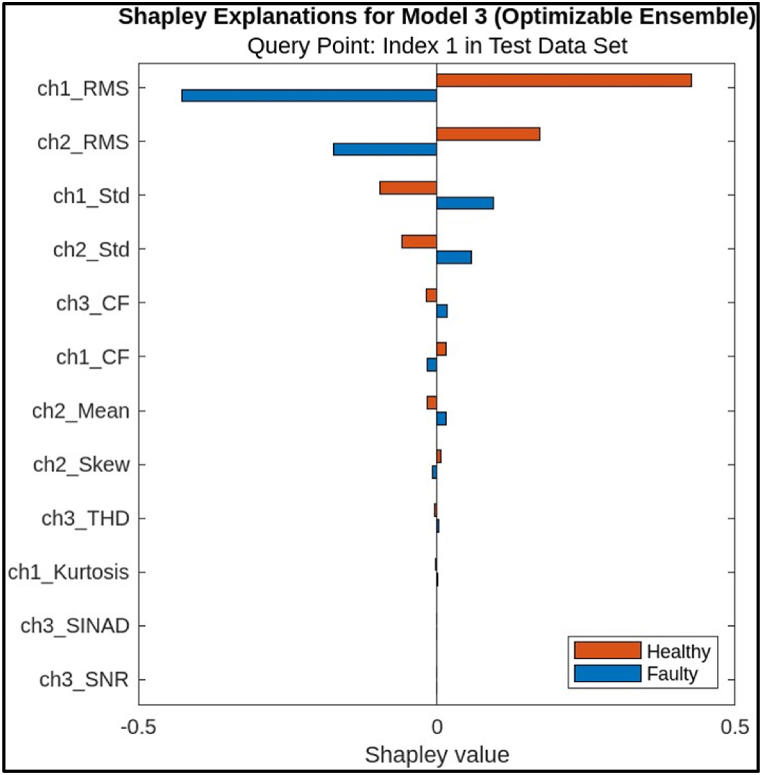
Shapley Explanations for ensemble Model.

Shapley values are calculated by permuting all possible feature orderings and measuring each feature's contribution to the prediction under each order. This approach is designed to be robust to how the features are correlated with each other. The bar chart, Fig. 16, visually depicts the Shapley values for each predictor, with the x-axis representing the Shapley value and the y-axis enumerating the predictors. A positive Shapley value indicates that a feature contributes to the prediction of a faulty condition, while a negative value implies a contribution to the prediction of a healthy condition. The length of the bar represents the magnitude of the feature's impact on the prediction.

Table 3, provides a numerical representation of the same information, listing each predictor, its corresponding value for the query point (Index 1 in the test dataset), and its associated Shapley value for both faulty and healthy conditions (see Table 4).

**Table 3:** Predictor & Shapley Value of features.

Predictor	Predictor Value	Shapley Value	
		Faulty	Healthy
ch1_RMS	0.2675	−0.4268	0.4268
ch2_RMS	3.0043	−0.1731	0.1731
ch1_Std	0.001548	0.0947	−0.0947
ch2_Std	0.01505	0.0587	−0.0587
ch3_CF	1.0078	0.0177	−0.0177
ch1_CF	1.0101	−0.016	0.016
ch2_Mean	−3.0043	0.0159	−0.0159
ch2_Skew	−0.01474	−0.0065	0.0065
ch3_THD	−1.7877	0.0029	−0.0029
ch1_Kurtosis	1.7454	0.0025	−0.0025
ch3_SINAD	23.766	0	0
ch3_SNR	27.7775	0	0

**Table 4:** Assessment of different algorithms employed for classifying features.

Sr. No.	Model Type	Status	Accuracy % (Validation)	Total Cost (Validation)	Accuracy % (Test)	Total Cost (Test)
1	Efficient Linear	Tested	96.22	388	96.34	89
2	Decision Tree	Tested	99.80	16	99.81	4
3	Ensemble	Tested	99.96	4	**100**	0
4	Neural Network	Tested	99.94	5	99.92	2

From the visualization and table, it is evident that the most influential predictor in this instance is ‘ch2_RMS’ with a substantial positive Shapley value of 0.4268 for the faulty condition and a negative value of −0.4268 for the healthy condition. This implies that a higher value of ‘ch2_RMS’ significantly increases the likelihood of the model predicting a faulty condition, while a lower value increases the likelihood of predicting a healthy condition. Other notable predictors with considerable impact on the prediction include ‘ch1_RMS’, ‘ch1_Std’, and ‘ch2_Std’, all exhibiting positive Shapley values for the faulty condition. In contrast, predictors like ‘ch3_CF’, ‘ch1_CF’, and ‘ch2_Mean’ show minimal impact on the prediction, with near-zero Shapley values for both conditions. This suggests that these features have limited influence on the model's decision-making process for this specific query point.

Hence, the analysis of the Shapley values reveals the key predictors contributing to the fault diagnosis of the CNC Hobbing cutter using the ensemble model. ‘ch2_RMS’ emerges as the most influential feature, with a substantial impact on differentiating between faulty and healthy conditions. Understanding the contributions of these features aided in developing strategies for fault prevention and maintenance.

#### 5.3.1 Justification of feature selection

The most efficient features, as identified by these methods, are those that significantly influence the classification of faulty and healthy conditions of the CNC hobbing cutter. In the LIME analysis, features such as 'ch1_CF' (Crest Factor) and 'ch1_Std' (Standard Deviation) exhibit the highest impact, with negative values indicating their critical role in identifying faults. Similarly, Shapley values highlight features like 'ch2_RMS' (Root Mean Square) as major contributors to the prediction of faulty conditions, with a substantial positive impact. As for the possibility of contradicting results between LIME and Shapley, it is important to recognize that these methods provide complementary perspectives. LIME is more localized and focuses on individual predictions, while Shapley offers a global view of feature importance. While discrepancies may occur due to their differing methodologies, the overall consistency in feature ranking across both methods suggests that they are in alignment, with the most influential features being consistently identified. Thus, no significant contradictions were observed in their results for the given features, affirming their reliability.

### 5.4 Feature classification

The research work proposed different methods for classifying the health of hobbing cutters. Tree-based classifiers, specifically Ensemble, Efficient Linear, and Decision Tree algorithms, achieved the best results. To find the most effective method among these tree-based classifiers, the algorithms were compared with their performance. The algorithms were tested using 20 % data. A technique called 10-fold cross-validation was also used for further evaluation. Then a confusion matrix employed to visualize how well each method distinguished between different hobbing cutter conditions [28].

**Fig. 17:**
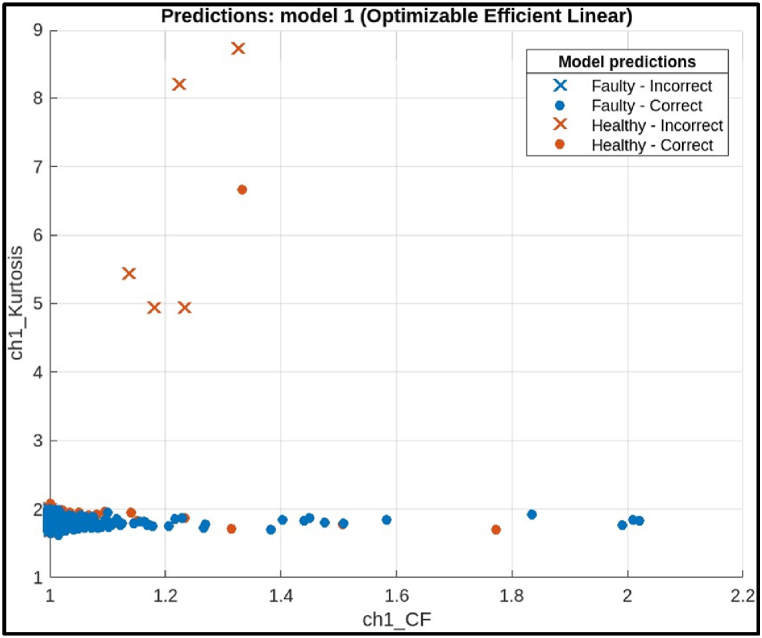
Scatter plot of Efficient Linear Model.

i) **Efficient Linear Classifier:** The scatter plot in Fig. 17 shows the performance of an efficient linear model for classifying hobbing cutter faults based on the "ch1_sigstats_CrestFactor" feature, which measures the peakiness of the vibration signal. The x-axis represents the crest factor, and the y-axis shows classification results (healthy vs. faulty). Blue points are classified as healthy, and red points as faulty. While red points tend to cluster on the right (*higher* crest factor), there is significant overlap with the blue points, indicating weak correlation. This suggests that the model's classification accuracy is limited when using crest factor alone.

**Fig. 18:**
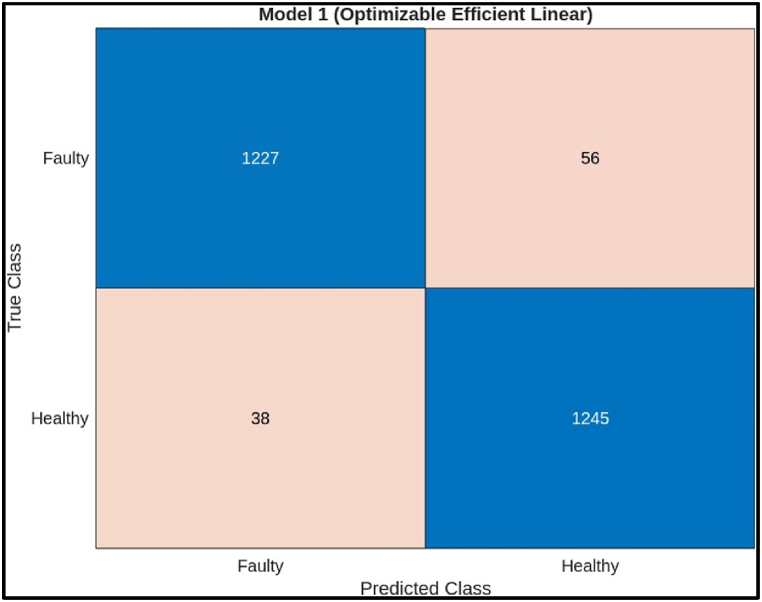
Confusion matrix of efficient linear Model.

The confusion matrix on test samples for the Efficient Linear model classifying CNC Hobbing Cutters as shown in Fig. 18. The calculation of its accuracy, precision, recall, and F1 score are shown below.

● *Classes:* The model classifies the hobbing cutters into two classes: Faulty and Healthy.
● *True Positives (TP):* The model correctly classified 1227 faulty samples.
● *False Negatives (FN):* The model incorrectly classified 56 faulty samples as healthy.
● *False Positives (FP):* The model incorrectly classified 38 healthy samples as faulty.
● ***True Negatives (TN):*** The model correctly classified 1245 healthy samples.

Calculations.

● *Accuracy:*$\frac{\left(TP+TN\right)\;}{Total}$ = $\frac{\left(1227+1245\right)\;}{\left(1227+56+38+1245\right)\;}$ = $\frac{2472}{2566\;}$ = 0.9634 (or 96.34 %)
● *Precision:*$\frac{TP\;}{\left(TP+FP\right)\;}$ = $\frac{1227}{\left(1227+38\right)\;}$ = $\frac{1227}{1265}=$ 0.97 (or 97.00 %)
● *Recall:*$\frac{TP\;}{\left(TP+FN\right)\;}$ = $\frac{1227}{\left(1227+56\right)\;}$ = $\frac{1227}{1283\;}$ = 0.9563 (or 95.63 %)
● *F1 Score:*$\frac{2\;X\;\left(Precision\;X\;Recall\right)\;}{\left(Precision+Recall\right)\;}$ = $\frac{2\;X\;\left(0.97\;X\;0.9563\right)\;}{\left(0.97+0.9563\right)\;}$ = $\frac{1.855}{1.946}$ = 0.9532 (or 95.32 %)

**Fig. 19:**
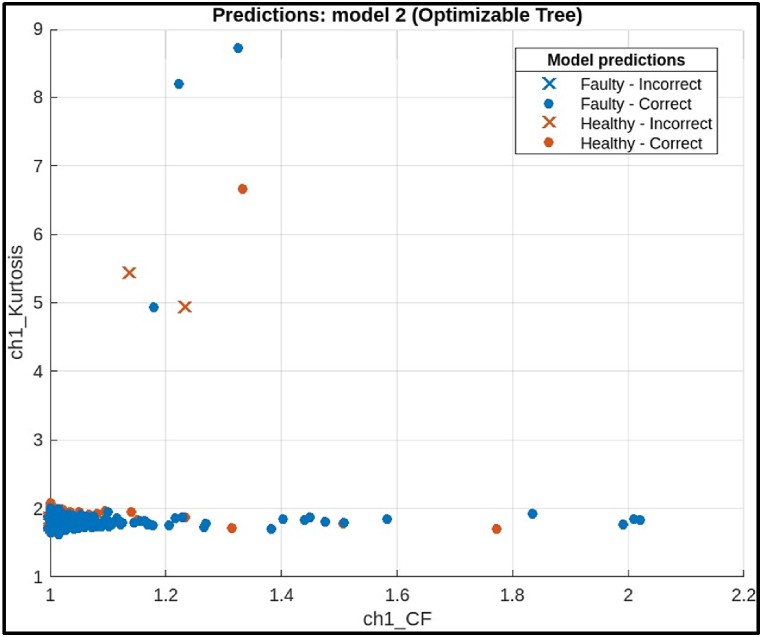
Scatter plot of Decision Tree Model.

The Efficient Linear model achieved a high accuracy of 96.33 %, indicating it correctly classified most of the hobbing cutters. It also has precision (97 %) and recall (95.63 %) is slightly lower. The model missed classifying 56 faulty hobbing cutters, which could be crucial depending on the application. The F1 score (95.32 %) captures both precision and recall, offering a comprehensive assessment of the model's effectiveness.

ii) **Decision Tree Classifier:** The scatter plot in Fig. 19 shows the performance of a decision tree model for classifying hobbing cutter faults using statistical features from channel 1. The x-axis likely represents the predicted fault class, and the y-axis represents the actual fault class. Blue points indicate correct classifications, while red points show misclassifications. Ideally, most points should be blue, with few red points. However, the plot shows a mismatch, with only 9 data points (3 blue, 6 red), making it difficult to assess the model's overall performance.

**Fig. 20:**
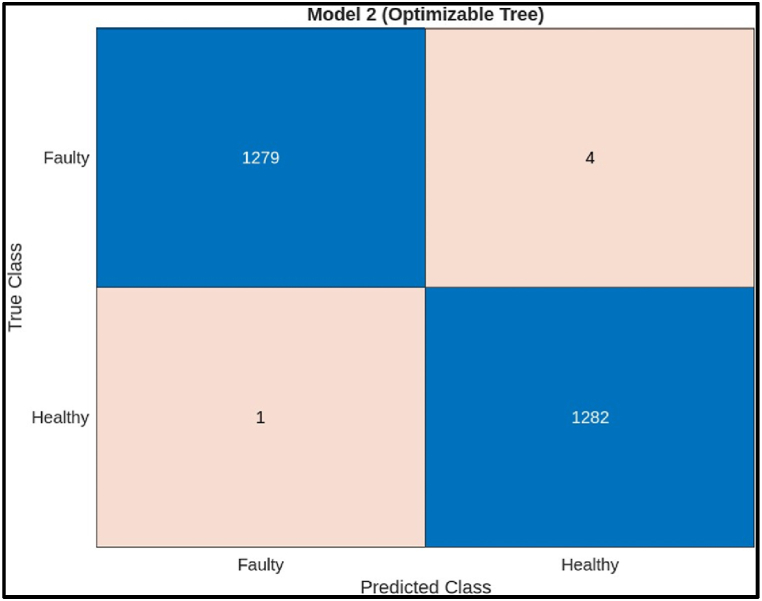
Confusion matrix of decision tree Model.

The confusion matrix on test samples for the decision tree model classifying CNC Hobbing Cutters as shown in Fig. 20, along with calculating its accuracy, precision, recall, and F1 score. The confusion matrix shows the following.

● *Classes:* The model classifies the hobbing cutters into two classes: Faulty and Healthy.
● *True Positives (TP):* The model correctly classified 1280 healthy samples.
● *False Negatives (FN):* The model incorrectly classified 4 faulty samples as healthy.
● *False Positives (FP):* The model incorrectly classified 1 healthy sample as faulty.
● *True Negatives (TN):* The model correctly classified 1282 faulty samples.

Calculations.

● *Accuracy:*$\frac{\left(TP+TN\right)\;}{Total}$ = $\frac{\left(1279+1282\right)\;}{\left(1279+4+1+1282\right)\;}$ = $\frac{2561}{2566\;}$ = 0.9981 (or 99.81 %)
● *Precision:*$\frac{TP\;}{\left(TP+FP\right)\;}$ = $\frac{1279}{\left(1279+1\right)\;}$ = $\frac{1279}{1280\;}$ = 0.9992 (or 99.92 %)
● *Recall:*$\frac{TP\;}{\left(TP+FN\right)\;}$ = $\frac{1279}{\left(1279+4\right)\;}$ = $\frac{1279}{1283\;}$ = 0.9968 (or 99.68 %)
● *F1 Score:*$\frac{2\;X\;\left(Precision\;X\;Recall\right)\;}{\left(Precision+Recall\right)\;}$ = $\frac{2\;X\;\left(0.9992\;X\;0.9968\right)\;}{\left(0.9992+0.9968\right)\;}$ = 0.9980 (or 99.80 %)

**Fig. 21:**
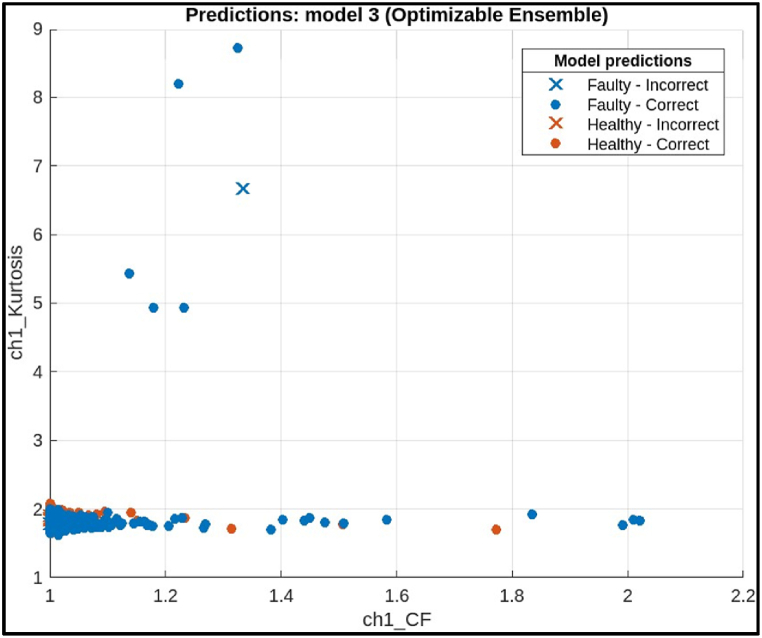
Scatter plot of Ensemble Model.

The decision tree model has a very high accuracy (99.80 %) on this test data. This means that the model was very successful in correctly classifying the health of the hobbing cutters. It also has a high precision (99.92 %) and recall (99.68 %), indicating that the model rarely makes mistakes in either classifying a healthy hobbing cutter as faulty (high precision) or missing a faulty hobbing cutter (high recall). The F1 score (99.80 %) is a harmonic mean between precision and recall, providing a balanced view of the model's performance.

ii) **Ensemble Classifier:** The scatter plot in Fig. 21 illustrates the performance of an ensemble model for classifying hobbing cutter faults based on statistical features from Channel 1. The x-axis represents the predicted fault class, and the y-axis represents the actual class. Blue points indicate correct classifications (healthy or faulty), while red points show misclassifications. The model performs well, with mostly blue points, suggesting minimal errors. It is more accurate at classifying healthy cutters, as evidenced by the concentration of blue points near the diagonal line, indicating better performance for healthy classifications.

**Fig. 22:**
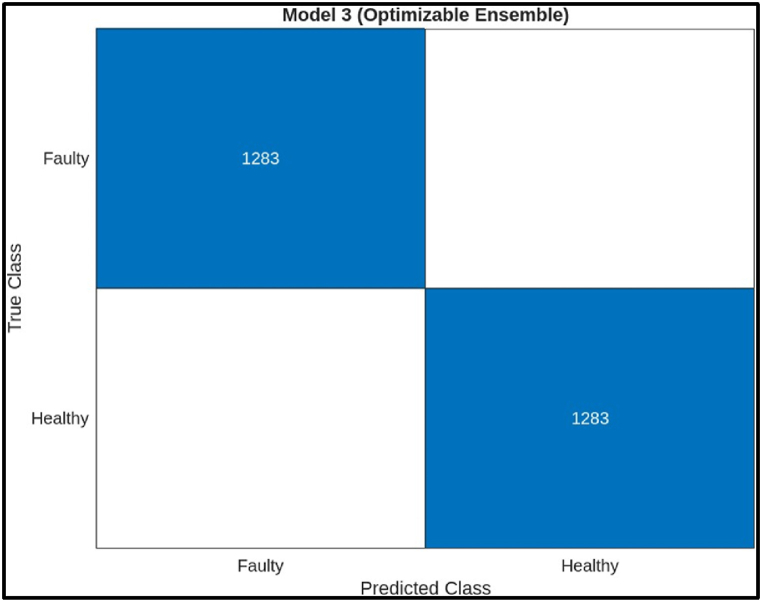
Confusion matrix of ensemble Model.

The confusion matrix on test samples for the Ensemble model classifying CNC Hobbing Cutter as shown in Fig. 22, along with calculating its accuracy, precision, recall, and F1 score. The confusion matrix shows the following.

● *Classes:* The model classifies the hobbing cutters into two classes: Faulty and Healthy.
● *True Positives (TP):* The model correctly classified 1283 healthy samples.
● *False Negatives (FN):* The model incorrectly classified 0 healthy samples as faulty.
● *False Positives (FP):* The model incorrectly classified 0 faulty samples as healthy.
● *True Negatives (TN):* The model correctly classified 1283 faulty samples.

Calculations.

● *Accuracy:*$\frac{\left(TP+TN\right)\;}{Total}$ = $\frac{\left(1283+1283\right)\;}{2566\;}$ = 1 (or 100 %)
● *Precision:*$\frac{TP\;}{\left(TP+FP\right)\;}$ = $\frac{1283}{\left(1283+0\right)\;}$ = 1 (or 100 %) **Perfect precision**
● *Recall:*$\frac{TP\;}{\left(TP+FN\right)\;}$ = $\frac{1283}{\left(1283+0\right)\;}$ = 1 (or 100 %) Perfect recall
● *F1 Score:*$\frac{2\;X\;\left(Precision\;X\;Recall\right)\;}{\left(Precision+Recall\right)\;}$ = $\frac{2\;X\;\left(1\;X\;1\right)\;}{\left(1+1\right)\;}$ = 1 (or 100 %)

**Fig. 23:**
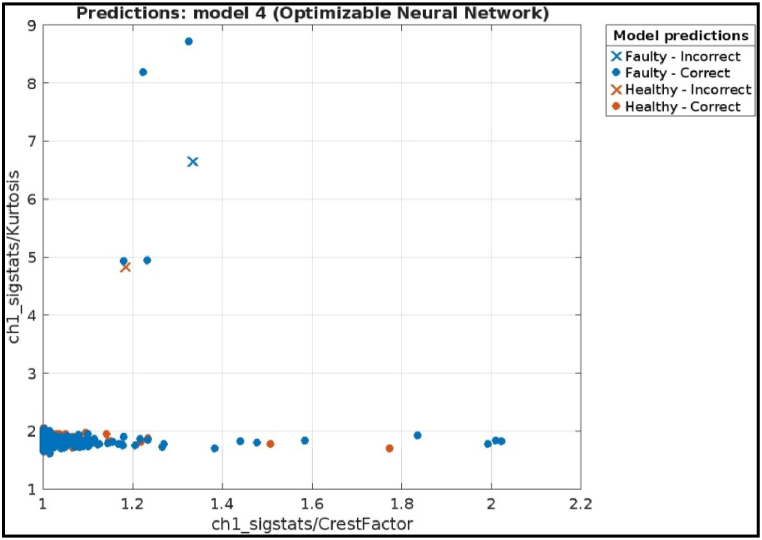
Scatter plot of Deep Neural Network.

The ensemble model achieved perfect scores on all metrics: accuracy, precision, recall, and F1 score. This indicates an ideal scenario where the model made no mistakes in classifying the hobbing cutter.

iv) **Deep Neural Network:** The plot given in Fig. 23 visualizes the predictions of the optimized neural network model for fault diagnosis in a CNC hobbing cutter, comparing two features: Crest Factor (x-axis) and Kurtosis (y-axis). Correctly classified healthy and faulty samples are marked with orange circles and blue crosses, respectively, while misclassified samples are indicated by distinct symbols. The distribution shows that the model effectively separates healthy and faulty conditions based on these features, with most data points clustered correctly. The few misclassifications suggest minor overlaps in feature values.

**Fig. 24:**
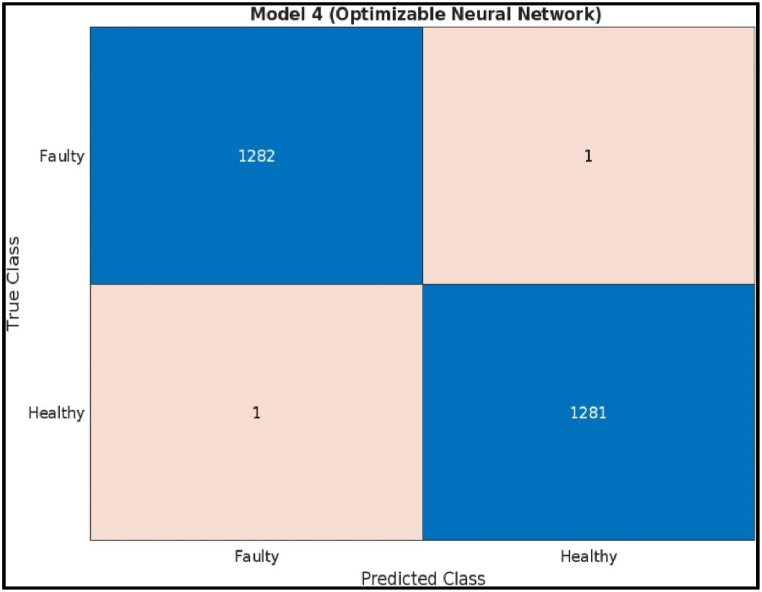
Confusion matrix of deep neural network.

The confusion matrix on test samples for the Deep Neural Network model classifying CNC Hobbing Cutter as shown in Fig. 24, along with calculating its accuracy, precision, recall, and F1 score. The confusion matrix shows the following.

● *Classes:* The model classifies the hobbing cutters into two classes: Faulty and Healthy.
● *True Positives (TP):* The model correctly classified 1282 healthy samples.
● *False Negatives (FN):* The model incorrectly classified 1 healthy sample as faulty.
● *False Positives (FP):* The model incorrectly classified 1 faulty sample as healthy.
● *True Negatives (TN):* The model correctly classified 1282 faulty samples.

Calculations.

● *Accuracy:*$\frac{\left(TP+TN\right)\;}{Total}$ = $\frac{\left(1282+1282\right)\;}{2566\;}$ = 0.9992 (or 99.92 %)
● *Precision:*$\frac{TP\;}{\left(TP+FP\right)\;}$ = $\frac{1282}{\left(1282+1\right)\;}$ = 0.9992 (or 99.92 %)
● *Recall:*$\frac{TP\;}{\left(TP+FN\right)\;}$ = $\frac{1282}{\left(1282+1\right)\;}$ = 0.9992 (or 99.92 %)
● *F1 Score***:**$\frac{2\;X\;\left(Precision\;X\;Recall\right)\;}{\left(Precision+Recall\right)\;}$ = $\frac{2\;X\;\left(0.9992\;X\;0.9992\;\right)\;}{\left(0.9992+0.9992\;\right)\;}$ = 0.9992 (or 99.92 %)

The Efficient Linear model achieved a high accuracy of 99.92 %, indicating it correctly classified most of the hobbing cutters. It also has precision (99.92 %) and recall (99.92 %) is slightly lower. The model missed classifying 1 faulty hobbing cutters, which could be crucial depending on the application. The F1 score (99.92 %) captures both precision and recall, offering a comprehensive assessment of the model's effectiveness.

### 5.5 Receiver Operating Characteristic (ROC) curve

A Receiver Operating Characteristic (ROC) Curve of a Decision Tree model, Ensemble Model and Efficient linear for fault diagnosis of a CNC Hobbing Cutter are shown below. An ROC curve is a visual tool used to assess the model performance in classifying things into two categories. It plots the rate of correctly identifying true positives (TPR) on the vertical axis against the rate of incorrectly identifying negatives as positives (FPR) on the horizontal axis.

● *TPR (True Positive Rate):* This is the proportion of correctly classified positive cases (correctly identified faulty cutters). A high TPR is desirable.
● *FPR (False Positive Rate):* This is the proportion of incorrectly classified negative cases (healthy cutters identified as faulty). A low FPR is desirable.

In the ideal scenario, a perfect classifier would achieve a TPR of 1 (all faulty cutters correctly identified) and an FPR of 0 (no healthy cutters misclassified). This would be represented by a point in the upper left corner of the ROC Curve.

**Fig. 25:**
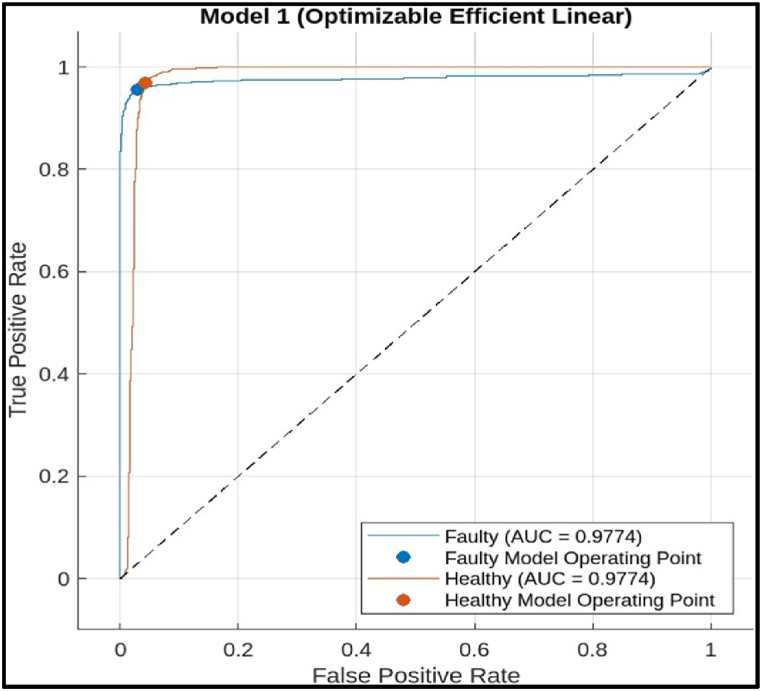
Roc of efficient linear Model.
ii) Decision Tree Model: The ROC curve in Fig. 26 shows a good performance for the decision tree model. The curve goes close to the upper left corner**;** this indicates that the model has achieved a high TPR with a low FPR. In other words, it has correctly classified most faulty cutter while producing few false alarms. The AUC (Area under the Curve) value is high (0.9981)**.** AUC reflects a model's overall performance, with values closer to 1 signifying better results. An AUC of 0.9981 suggests the decision tree model has a very good ability to distinguish between faulty and healthy hobbing cutter.

i) Efficient Linear Model: The ROC curve in Fig. 25 shows a good performance for the efficient linear model. The curve goes close to the upper left corner**;** this indicates that the model achieved a high TPR with a low FPR. In other words, it has correctly classified most faulty cutter while producing few false alarms. The model performed well, achieving a high AUC of 0.9774 (Area under the curve). A value closer to 1 indicates better performance. An AUC of 0.9774 suggests the efficient linear model has a very good ability to distinguish between faulty and healthy hobbing cutter. The ROC Curve in the figure indicates that the decision tree model is a promising tool for fault diagnosis of CNC hobbing cutter. It has a high AUC, suggesting good overall performance in distinguishing between faulty and healthy cutters.

The ROC Curve in the figure indicates that the decision tree model is a promising tool for fault diagnosis of CNC hobbing cutter. It has a high AUC, suggesting good overall performance in distinguishing between faulty and healthy cutters.

iii) Ensemble Model: The ROC curve in Fig. 27 shows an almost perfect performance for the ensemble model.

**Fig. 26:**
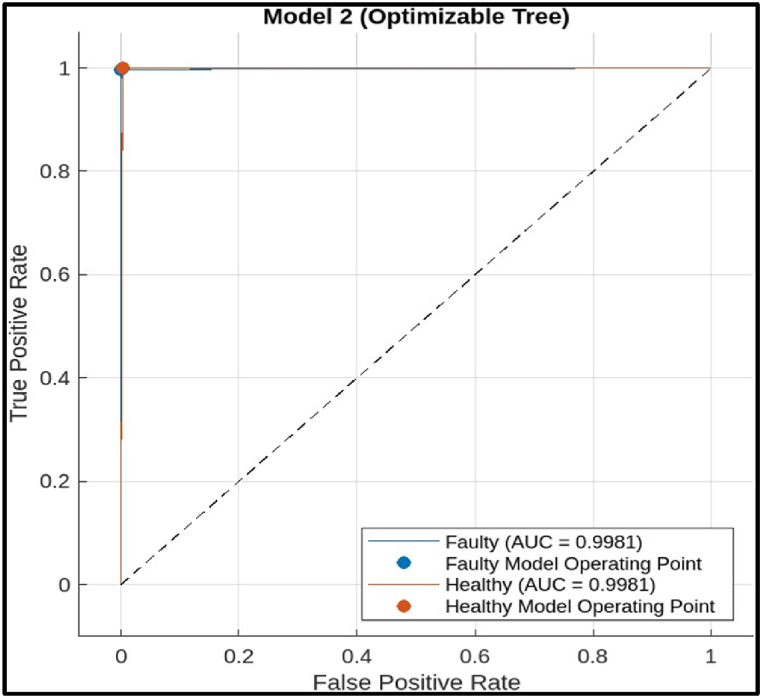
Roc of decision tree Model.

**Fig. 27:**
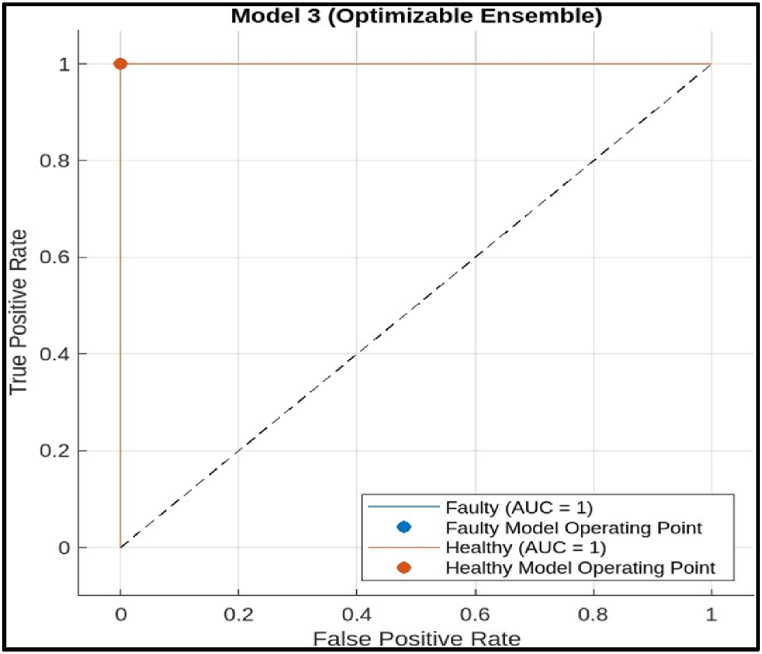
Roc of ensemble Model.

**Fig. 28:**
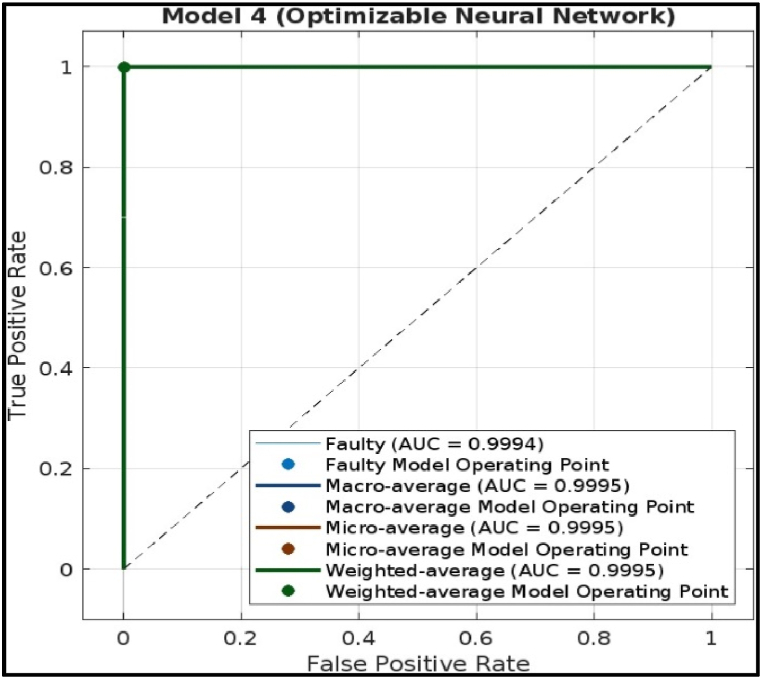
ROC of Deep Neural Network model.

The curve suggests the curve goes to the upper left corner and overlaps with the axes. This indicates that the model has achieved a perfect TPR (1) with a zero FPR (0). In other words, it correctly classifying all faulty cutters (100 % TPR) while producing no false alarms (0 % FPR). The ROC Curve in the figure suggests that the Ensemble model achieved a perfect classification on the test data.

iv) Deep Neural Network model: The ROC curve demonstrates in Fig. 28 that the Deep Neural Network model achieves near-perfect classification performance for fault diagnosis of CNC hobbing cutters, with AUC values exceeding 0.9994 across all metrics (Faulty, Macro, Micro, and Weighted averages). The model exhibits a high True Positive Rate (TPR) and a very low False Positive Rate (FPR), ensuring accurate detection of both faulty and healthy cutters with minimal misclassification. This highlights the model's exceptional reliability and suitability for industrial fault diagnosis.

### 5.6 Selection of best model based on test accuracy

The comparison table presents the performance of four machine learning models—Efficient Linear, Decision Tree, Ensemble, and Neural Network—used for classifying features in CNC hobbing cutter fault diagnosis. The Accuracy % (Validation) and Accuracy % (Test) columns indicate how well each model performed on the validation and test datasets, respectively, while the Total Cost columns show the computational cost associated with each model during validation and testing.

• *Efficient Linear* achieved an accuracy of 96.22 % on validation and 96.34 % on the test set, with moderate computational costs of 388 for validation and 89 for testing.
• *Decision Tree* performed very well with an accuracy of 99.80 % (validation) and 99.81 % (test), but its validation cost (16) was higher compared to the Ensemble and Neural Network models.
• *Ensemble* model exhibited the best performance with 99.96 % accuracy on the validation set and 100 % accuracy on the test set, alongside the lowest computational cost (4 for validation and 0 for testing).
• *Neural Network* achieved almost identical results to the Ensemble model in terms of accuracy (99.94 % on validation and 99.92 % on test) but required slightly higher computational costs (5 for validation and 2 for testing).

The Ensemble model is the best choice for this application due to its highest test accuracy (100 %) and lowest total cost. This model provides optimal classification performance with minimal computational overhead, making it ideal for real-time fault diagnosis in CNC hobbing cutters. Its superior test performance suggests that it generalizes better to unseen data compared to the other models. Among recently published studies (Table 5), the current framework demonstrates the best classification accuracy.

**Table 5:** Reliability of tool condition assessment in published investigations.

Sr. No.	Classifier	Nature of Signal	Type of features	Accuracy in %	Reference
1	Ensemble	Time-domain	Statistical & histogram	100.00	**Proposed Model**
2	Random forest based on K-nearest neighbor	Time-domain wavelet threshold de-noising	Statistical	98.74	Zhongling Xue et al. (2023)
3	Best first tree	Time-domain	Statistical	97.00	A. Patange et al. (2021)
4	SVM	Time-frequency	Holder Exponents	90.80	Zhou, Yang et al. (2020)
5	Calibration-model	Time series	Statistical	90.00	Rui Liu et al. (2020)
6	ANN	Time series	Averaging and Weighted-Averaging	<90.00	Kehua Guo et al. (2020)
7	SVM	Time-domain	Statistical	91.83	Jiayu Ou et al. (2019)
8	Continuous Hidden Markov Models	Time-domain de-noised by wavelet	Statistical	91.20	Zhengyou Xie et al. (2019)
9	SVM	Time-frequency	Holder Exponents	86.20	Zhou, Guo et al. (2019)
10	SVR	Time-frequency	Wavelet packets	85.50	Fatemeh Aghazadeh et al. (2018)
11	Multilayer Perceptron	Time-domain	Statistical & histogram	82.50	N. Gangadhar et al. (2018)
12	Decision tree J48	Time-domain de-noised by wavelet	Statistical	82.41	Ravikumar. S. et al. (2018)

## 6 Future Scope

While this study establishes a robust fault diagnosis framework, it is essential to address certain limitations and explore future research opportunities.

• Expanding the dataset to include real-world machining environments with varying conditions and fault types for enhanced model robustness.
• Incorporating multi-modal sensor data, such as acoustic and temperature signals, to improve diagnostic accuracy.
• Exploring transfer learning and online learning methods for rapid adaptation to different machines and tooling configurations.
• Developing user-friendly visualization tools for operators to interpret diagnostics easily, fostering wider industrial adoption.

## 7 Conclusions

This research presents a comprehensive study on fault diagnosis for CNC hobbing cutters using machine learning techniques, leveraging three-axis vibration data for condition monitoring. The proposed framework employs advanced statistical feature extraction and machine learning algorithms to accurately classify cutter health conditions under healthy and faulty states. The highlights of this work are as follows.

• *Optimal Model Performance:* Among the tested models, the Ensemble classifier demonstrated outstanding performance, achieving a perfect test accuracy of 100 %. It surpassed alternatives like Decision Trees, Efficient Linear Models, and Deep Neural Networks in accuracy and computational efficiency, making it the most suitable choice for fault diagnosis in CNC hobbing operations. This result underscores the Ensemble model's superior ability to generalize to unseen data, ensuring reliability in diverse machining conditions.
• *Explainable AI Integration:* The study incorporated Explainable AI (XAI) techniques such as LIME and Shapley values, providing interpretable insights into the key features influencing model predictions. Features like Root Mean Square (RMS), Crest Factor, and Standard Deviation emerged as significant contributors to the classification, enhancing the transparency and trustworthiness of the diagnostic process.
• *Robust Methodology:* The methodology included data pre-processing, advanced feature engineering, hyperparameter optimization through Bayesian techniques, and rigorous model evaluation using confusion matrices, Receiver Operating Characteristic (ROC) curves, and other performance metrics. This structured approach ensured high accuracy and minimized overfitting, addressing the challenges of non-stationary vibration signals.
• *Industrial Applicability:* The research emphasizes the practical implications of machine learning in real-time fault diagnosis, reducing downtime and improving operational efficiency in the machining industry. The use of tri-axial vibration data aligns with industrial sensor setups, and the results demonstrate the feasibility of deploying AI-driven diagnostic systems in production environments.

This research validates the transformative potential of machine learning in predictive maintenance for CNC machines, offering a pathway toward reducing the downtime, enhanced productivity, cost savings, and quality assurance in the manufacturing sector. By bridging the gap between advanced AI methodologies and practical applications, this work sets a benchmark for future innovations in cutting tool condition monitoring.

## CRediT authorship contribution statement

**Nagesh Tambake:** Writing – review & editing, Writing – original draft, Visualization, Validation, Resources, Investigation, Formal analysis, Data curation, Conceptualization. **Bhagyesh Deshmukh:** Writing – review & editing, Writing – original draft, Visualization, Validation, Supervision, Software, Funding acquisition, Formal analysis, Conceptualization. **Sujit Pardeshi:** Writing – original draft, Visualization, Resources, Project administration, Investigation, Funding acquisition. **Sachin Salunkhe:** Writing – review & editing, Writing – original draft, Software, Resources, Project administration, Investigation, Formal analysis. **Robert Cep:** Writing – review & editing, Writing – original draft, Visualization, Supervision, Resources, Data curation. **Emad Abouel Nasr:** Validation, Supervision, Resources, Methodology, Investigation, Funding acquisition, Formal analysis.

## Data Availability

All data that support the findings of this study are included within this article.

## Funding

The authors also extend their appreciation to King Saud University for funding the publication of this work through Researchers Supporting Project number (RSP2025R164), King Saud University, Riyadh, Saudi Arabia. This article was co-funded by the European Union under the REFRESH – Research Excellence For Region Sustainability and High-tech Industries project number CZ.10.03.01/00/22_003/0000048 via the Operational Programme Just Transition and has been done in connection with project Students Grant Competition SP2024/087, Specific Research of Sustainable Manufacturing Technologies“ financed by the Ministry of Education, Youth and Sports and Faculty of Mechanical Engineering VŠB-TUO. Article has been done in connection with project Students Grant Competition SP2024/087 „Specific Research of Sustainable Manufacturing Technologies“ financed by the Ministry of Education, Youth and Sports and Faculty of Mechanical Engineering VŠB-TUO.

## Declaration of competing interest

The authors declare that they have no known competing financial interests or personal relationships that could have appeared to influence the work reported in this paper.
